# The microRNA‐204‐5p inhibits APJ signalling and confers resistance to cardiac hypertrophy and dysfunction

**DOI:** 10.1002/ctm2.693

**Published:** 2022-01-21

**Authors:** Ravinder Reddy Gaddam, Young‐Rae Kim, Julia S. Jacobs, Jin‐Young Yoon, Qiuxia Li, Angela Cai, Hamsitha Shankaiahgari, Barry London, Kaikobad Irani, Ajit Vikram

**Affiliations:** ^1^ Department of Internal Medicine Carver College of Medicine University of Iowa Iowa City Iowa USA

**Keywords:** apelin‐receptor, aplnr, ERK1/2, heart failure, microRNA‐204, noncoding RNAs

## Abstract

**Background:**

MicroRNAs regulate cardiac hypertrophy development, which precedes and predicts the risk of heart failure. microRNA‐204‐5p (miR‐204) is well expressed in cardiomyocytes, but its role in developing cardiac hypertrophy and cardiac dysfunction (CH/CD) remains poorly understood.

Methods: We performed RNA‐sequencing, echocardiographic, and molecular/morphometric analysis of the heart of mice lacking or overexpressing miR‐204 five weeks after trans‐aortic constriction (TAC). The neonatal rat cardiomyocytes, H9C2, and HEK293 cells were used to determine the mechanistic role of miR‐204.

**Results:**

The stretch induces miR‐204 expression, and miR‐204 inhibits the stretch‐induced hypertrophic response of H9C2 cells. The mice lacking miR‐204 displayed a higher susceptibility to CH/CD during pressure overload, which was reversed by the adeno‐associated virus serotype‐9‐mediated cardioselective miR‐204 overexpression. Bioinformatic analysis of the cardiac transcriptomics of miR‐204 knockout mice following pressure overload suggested deregulation of apelin‐receptor (APJ) signalling. We found that the stretch‐induced extracellular signal‐regulated kinase 1/2 (ERK1/2) activation and hypertrophy‐related genes expression depend on the APJ, and both of these effects are subject to miR‐204 levels. The dynamin inhibitor dynasore inhibited both stretch‐induced APJ endocytosis and ERK1/2 activation. In contrast, the miR‐204‐induced APJ endocytosis was neither inhibited by dynamin inhibitors (dynasore and dyngo) nor associated with ERK1/2 activation. We find that the miR‐204 increases the expression of ras‐associated binding proteins (e.g., Rab5a, Rab7) that regulate cellular endocytosis.

**Conclusions:**

Our results show that miR‐204 regulates trafficking of APJ and confers resistance to pressure overload‐induced CH/CD, and boosting miR‐204 can inhibit the development of CH/CD.

## Graphical abstract

miR‐204 regulates trafficking of APJ and confers resistance to pressure overload‐induced cardiac hypertrophy and cardiac dysfunction.

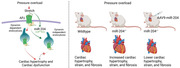

## 1 INTRODUCTION

Heart failure is a global health problem that affects >26 million people worldwide. In the United States alone, the prevalence of heart failure increased from 5.7 million for 2009–2012 to 6.5 million for 2011–2014.^1^ The heart failure incidence is expected to continue to rise with the increase in average age and survival following other cardiovascular disorders. The heart physiologically responds to mechanical stretch at a cellular level, but a sustained pressure overload during diseases like aortic stenosis or hypertension leads to heart failure. Recent studies demonstrate the critical role of noncoding RNAs (e.g., microRNAs, long noncoding RNAs) in cardiovascular disorders.^2^, ^3^ The microRNA‐204‐5p (miR‐204‐5p, referred to as miR‐204) is highly expressed in cardiomyocytes, and its deficiency increases the valvular osteogenic activity.^4^, ^5^ The expression of miR‐204 in cardiomyocytes exceeds that in noncardiomyocytes by 10 fold.^4^ Although no previous studies focused on the role of miR‐204 in the development of heart failure, the published miR expression profile demonstrates cardiac miR‐204 upregulation during heart failure.^6^, ^7^, ^8^, ^9^ The long noncoding RNA KCNQ1OT1 that acts as a sponge (binds to multiple copies of miR) for miR‐204, exacerbates the myocardial ischemia‐reperfusion injury in the mice.^10^, ^11^ Further, the inhalation of the general anaesthetic, sevoflurane, upregulated the miR‐204 expression in the heart and protected against myocardial ischemia‐reperfusion injury.^12^ The downregulation of the miR‐204 target‐gene translatome during pressure overload^13^ and the involvement of miR‐204 in valvular osteogenesis and myocardial ischemia‐reperfusion injury,^5^, ^10^, ^11^, ^12^ suggest a fundamental but yet unknown role for miR‐204 in the heart. In this study, we investigated the role of miR‐204 in the development of cardiac hypertrophy and cardiac dysfunction (CH/CD) using in vitro cyclic stretching of cardiomyocytes and in vivo pressure overload in mice. We also verified the human relevance by measuring the miR‐204 expression in the cardiac tissue of cardiomyopathy patients. Our results demonstrate that upregulation of cardiac miR‐204 during hypertrophic stress is a compensatory response, and miR‐204 intervention can rescue the pressure overload‐induced CH/CD. Our results also show that the miR‐204 governs the cellular trafficking of APJ, and the beneficial effects of miR‐204 depend on the inhibition of the maladaptive cardiac signalling of APJ.

## 2 RESULTS

### 2.1 miR‐204 inhibits the hypertrophic response of cardiomyocytes

**FIGURE 1:**
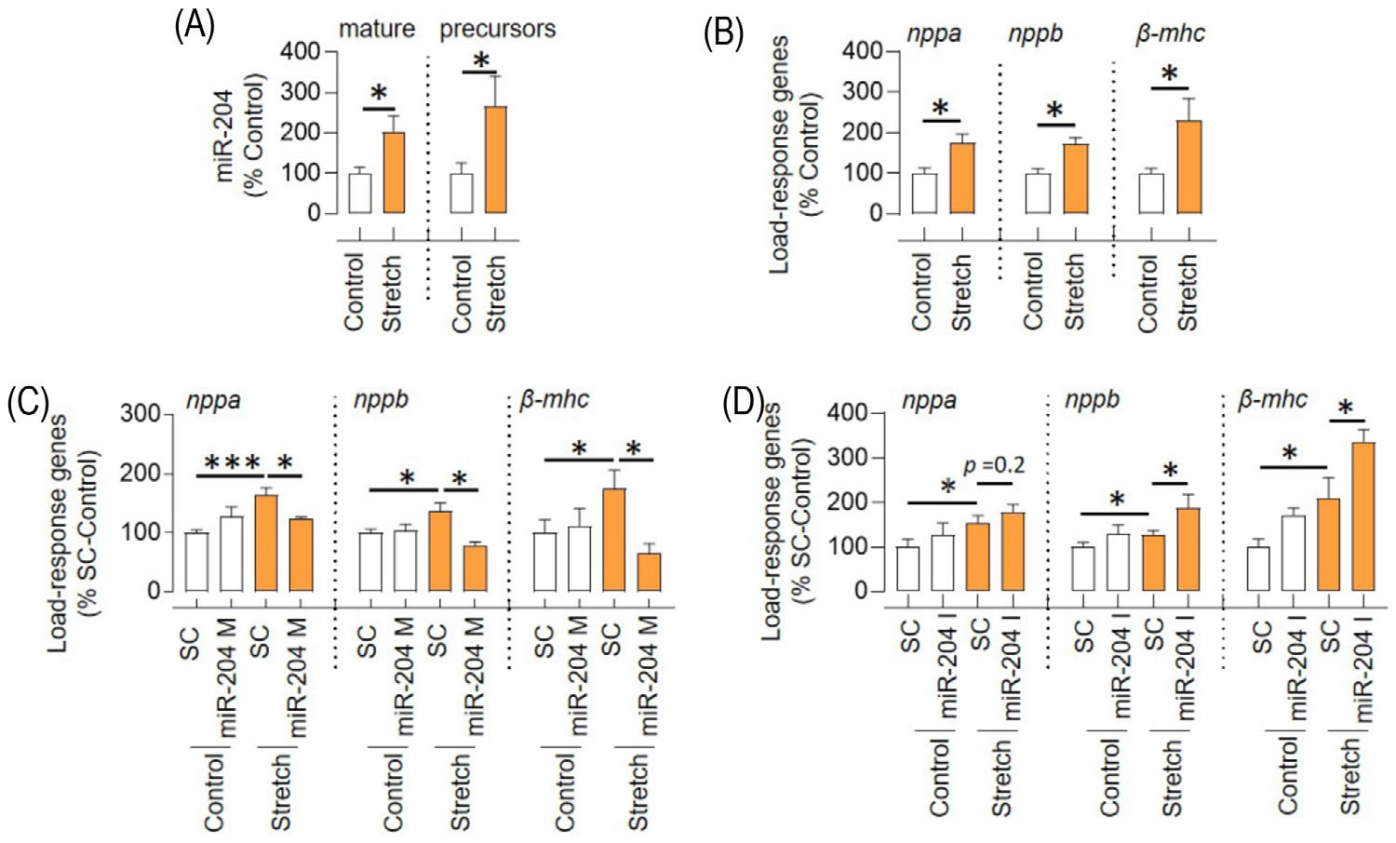
miR‐204 inversely regulates the stretch response of H9C2 cells. (A and B) The effect of 20% stretch (after 24 h) on the expression of (A) miR‐204 (mature and precursor) and (B) load‐response genes (*nppa, nppb* and *β‐mhc*) in H9C2 cells (*n* = 7). (C) The effect of miR‐204 mimic (miR‐204 M, 100 nM) on the expression of load‐response genes in H9C2 cells during stretching (*n* = 6). (D) The effect of miR‐204 inhibitor (miR‐204 I, 100 nM) on the expression of load‐response genes in H9C2 cells during stretching (*n* = 9). Data are shown as the mean, and error bars represent SEM. *p < .05 versus indicated group. SC, scrambled control; *nppa*, natriuretic peptide a*; nppb*, natriuretic peptide b; *β‐mhc*, beta‐myosin heavy chain

The H9C2 cells are myocytes derived from embryonic rat heart tissue. The response of H9C2 cells to hypertrophic factors is similar to that of the primary cardiomyocytes.^14^ We used H9C2 cells as an alternative to the primary cardiomyocytes. The H9C2 cells were stretched to 20% at 30 cycles/min for 24 h to recapitulate the effects of mechanical stretching. We found that this stretching induced an increase in the expression of miR‐204 (mature and precursor) and load‐response genes (*nppa*, *nppb* and *β‐mhc*) in H9C2 cells (Figure 1A,B and Figure S1A,B). The stretching of H9C2 cells for 24 h led to an increase in the cell size and was not associated with apoptosis as we did not observe any change in the levels of caspase‐3 (Figure S1C–E). The transcription factor STAT3 represses miR‐204 expression.^15^, ^16^, ^17^ Inhibition of STAT3 signalling in H9C2 cells with a dominant‐negative STAT3 (DNSTAT3) construct stimulated miR‐204 expression (Figure S1F,G). Moreover, the DNSTAT3 enhanced stretch‐induced miR‐204 in the H9C2 cells (Figure S1H), suggesting that stretch‐induced miR‐204 transcription involves STAT3. We determined the effect of angiotensin‐II that induces hypertrophy in H9C2 cells,^18^, ^19^ on miR‐204 expression, and found a significant increase in miR‐204 and load‐response genes expression (Figure S2A). To test the causal relationship between miR‐204 and the hypertrophic response of H9C2 cells, we determined the effect of miR‐204 mimic and inhibitor on the induction of load‐response genes during stretching. The transfection of miR‐204 mimic and inhibitor led to a significant increase and decrease in the miR‐204 expression in H9C2 cells, respectively (Figure S2B,C). The miR‐204 mimic abated while miR‐204 inhibitor augmented the stretch‐induced expression of load‐response genes, respectively (Figure 1C,D). Next, we verified the effects of miR‐204 in neonatal rat cardiomyocytes (NRCMs). The phenylephrine treatment led to an increase in the NRCMs’ cell size and expression of *nppa* and *nppb*, the effect that was aggravated by miR‐204 inhibition (Figure S1I–L). These data demonstrate that hypertrophic stress induces miR‐204 expression, and miR‐204 inhibits the stress‐response of cardiomyocytes.

### 2.2 miR‐204 regulates the development of CH/CD during pressure overload

**FIGURE 2:**
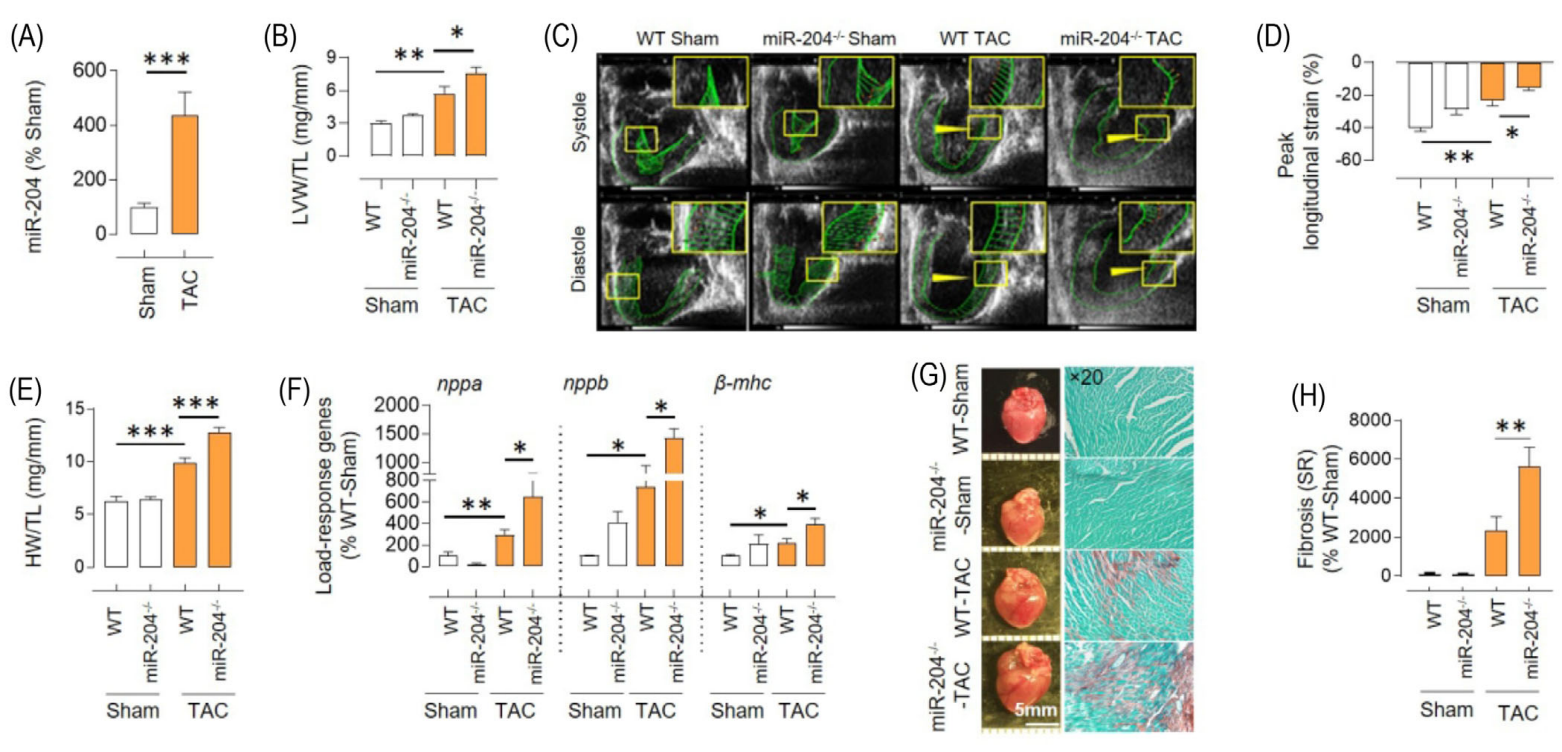
The absence of miR‐204 promotes pressure overload‐induced CH/CD. (A) The effect of trans‐aortic constriction (TAC)‐induced pressure overload on the expression of miR‐204‐5p in the heart of wild‐type (WT) mice (*n* = 7–13). (B) Echocardiography‐based assessment of left ventricular weight (LVW) normalised to tibia length (TL) in WT and miR‐204^–/–^ sham/TAC mice (*n* = 5–10). (C) Representative speckle trackings to measure the cardiac longitudinal strain at systole and diastole. A smaller horizontal green line indicates a higher strain. (D) Quantification of the peak longitudinal strain (%) (*n* = 5–10). (E) Heart weight (HW) normalised to tibia length (TL) in WT and miR‐204^–/–^ sham/TAC mice (*n* = 5–10). (F) Expression of hypertrophic markers (*nppa, nppb* and *β‐mhc*) in the left ventricle of WT and miR‐204^–/–^ sham/TAC mice (*n* = 5–10). (G) Representative images showing the effect of miR‐204 absence on cardiac enlargement and fibrosis 5 weeks after sham/TAC surgery. Sirius Red staining shows cardiac fibrosis (brown) at ×20 magnification. (H) Quantification of cardiac fibrosis. *n*(*N*) = 25(5). In (H), the replicates are shown as ‘n(N)’, where ‘*n*’ represents the number of fields and ‘*N*’ represents the number of mice. Data are shown as the mean, and error bars represent SEM. *p < .05, **p < .01, ***p < .001 versus indicated group. *nppa*, natriuretic peptide a; *nppb*, natriuretic peptide b; *β‐mhc*, beta‐myosin heavy chain

To determine the role of miR‐204 in vivo, we used trans‐aortic constriction (TAC) to induce pressure overload in mice with heterozygous (miR‐204^+/–^) and homozygous (miR‐204^–/–^) deletion of miR‐204 (Figure S3A). The whole‐body miR‐204 knockout mice had 10 bp deletion from the miR‐204‐5p region. miR‐204‐5p is the major strand of mature miR‐204, and miR‐204‐3p is the minor strand and has a much lower abundance than miR‐204‐5p.^20^ We found that the wild‐type (WT) mice had over 100‐fold higher miR‐204‐5p expression than the miR‐204‐3p, and miR‐204^–/–^ mice expressed approximately 40% lower miR‐204‐3p (Figure S3B). We did not notice any phenotypic difference in the cardiac parameters of WT, miR‐204^+/–^ and miR‐204^–/–^ mice at the baseline (Figure S3C–E). The functional and morphometric cardiac parameters of WT and miR‐204^–/–^ mice were assessed 5 weeks after TAC surgery. The pressure overload itself led to a significant increase in the expression of miR‐204 in the heart (Figure 2A). The time‐dependent study shows that pressure overload leads to an increase in miR‐204 as early as 2 weeks post TAC surgery and remains higher at 5 weeks after TAC surgery (Figure S3F). The two‐dimensional speckle tracking echocardiography‐derived myocardial strain is a sensitive marker of left ventricular dysfunction.^21^ We found a significant increase in the peak/global longitudinal strain, left ventricular weight and wall thickness, a higher end‐systolic/diastolic volume and a lower heart rate in miR‐204^–/–^ TAC mice compared to WT‐TAC mice, all indicating higher susceptibility to the CH/CD (Figure 2B–D, Table S1, Supporting Videos S1–S, 4). Per the echocardiographic data, we found an increase in the load‐response genes and enlargement of the heart in the miR‐204^–/−^ TAC mice (Figure 2E–G). In both WT‐TAC and miR‐204^–/–^ TAC mice, we observed a mild impairment in the ejection fraction. This could be because echocardiographic assessments were done in the early stages of cardiac remodelling or the use of 6J substrain of C57BL/6 mice, which develops a more robust hypertrophic phenotype but a milder cardiac dysfunction.^22^, ^23^ We analysed cardiac fibrosis using Sirius Red staining and found a significantly higher occurrence of fibrosis in the hearts of miR‐204^–/–^ TAC mice (Figure 2G,H). Moreover, we found increased expression of collagen genes (*Col1a1*, *Col1a2* and *Col3a1*), supporting that the mechanisms contributing to fibrosis were activated in the heart of miR‐204^–/–^ TAC mice (Figure S3G). In addition, the cross‐sectional area of cardiomyocytes was significantly higher in the heart of miR‐204^–/−^ TAC mice than in WT‐TAC mice (Figure S3H). The expression of load‐response genes and collagen genes were higher in the miR‐204^−/−^ Sham mice compared to the WT‐Sham (Figure 2F and Figure S3G), but we did not observe any CH/CD phenotype in miR‐204^–/−^ Sham mice compared to the WT‐Sham mice (Figure 2B–E). We found that the miR‐204^+/−^ were like WT‐TAC mice regarding miR‐204 expression in the heart and the CH/CD phenotype (Figure S4).

1 **Highlight**

1. MicroRNA‐204 is upregulated in the heart during cardiac stress, and it confers resistance to cardiac hypertrophy and cardiac dysfunction.
2. Cardioselective microRNA‐204 upregulation prevents the development of cardiac hypertrophy and cardiac dysfunction during pressure overload.
3. MicroRNA‐204 inhibits the ability of APJ to mediate maladaptive signalling by promoting its endocytosis via an alternative route, which contributes to the cardioprotective effects of microRNA‐204.

**FIGURE 3:**
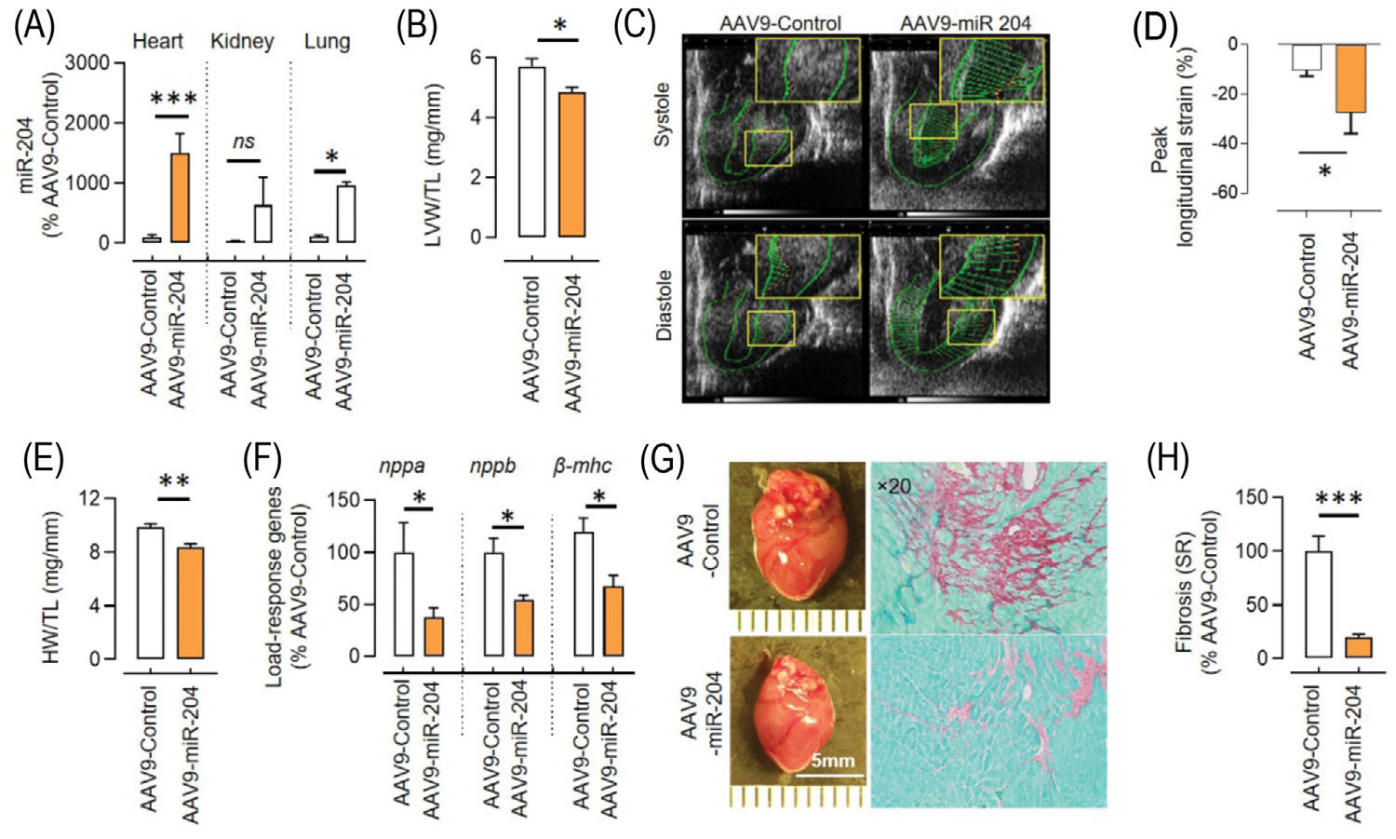
miR‐204 rescues pressure overload‐induced CH/CD. (A) Cardioselective miR‐204 upregulation by a single injection of AAV9‐miR‐204 (5 × 10^11^ viral genomes/mouse) 1 week before TAC surgery in miR‐204^–/–^ mice. Samples were collected 5 weeks after TAC surgery; control mice received the AAV9‐control virus (*n* = 5). (B) Echocardiography‐based assessment of left ventricular weight (LVW) normalised to tibia length (TL) in miR‐204^–/–^ TAC mice receiving either AAV9‐miR‐204 or AAV9‐control virus (*n* = 5). (C) Representative speckle trackings to measure the cardiac longitudinal strain at systole and diastole. A longer horizontal green line indicates a lower strain. (D) Quantification of the peak longitudinal strain (%) (*n* = 5). (E) Heart weight (HW) normalised to TL in miR‐204^–/–^ TAC mice receiving AAV9‐miR‐204 or AAV9‐control virus (*n* = 5). (F) Expression of load‐response genes in the left ventricle of miR‐204^–/–^ TAC mice receiving AAV9‐miR‐204 or AAV9‐control virus (*n* = 5). (G) Representative images showing the effect of cardioselective miR‐204 upregulation on cardiac enlargement and fibrosis during TAC. Sirius Red staining shows cardiac fibrosis (brown) at ×20 magnification. (H) Quantification of cardiac fibrosis. *n*(*N*) = 25(5). In (H), the replicates are shown as ‘*n*(*N*)’, where ‘*n*’ represents the number of fields and ‘*N*’ represents the number of mice. Data are shown as mean, and error bars represent SEM. *p < .05, **p < .01, ***p < .001 versus indicated group. *nppa*, natriuretic peptide a*; nppb*, natriuretic peptide b; *β‐mhc*, beta‐myosin heavy chain

Next, we investigated whether cardioselective overexpression of miR‐204 rescues the hypertrophic response in the miR‐204^–/–^ TAC mice. We used adeno‐associated virus serotype‐9 containing miR‐204 (AAV9‐miR‐204) to cardioselectively deliver miR‐204. A single injection of the AAV9‐miR‐204 virus through the jugular vein, cardioselectively increased miR‐204 expression in WT mice (Figure S5A). Next, we injected either AAV9‐miR‐204 or AAV9‐control virus in miR‐204^–/–^ mice 1 week before TAC surgery (Figure S5B,C), which led to a cardioselective increase in miR‐204 expression (Figure 3A). Five weeks after TAC surgery, we found a significant improvement in the peak/global longitudinal strain, left ventricular weight and wall thickness, end‐systolic/diastolic volume and heart rate in miR‐204^–/–^ TAC mice that received AAV9‐miR‐204, compared to those that received AAV9‐control virus (Figure 3B–D, Table S2). Per the echocardiographic data, we found a significant decrease in the load‐response genes and a rescue of cardiac enlargement in miR‐204^–/−^ TAC mice that received AAV9‐miR‐204 (Figure 3E–G). We stained cardiac sections with Sirius Red and found significantly lower fibrosis in the hearts of miR‐204^–/–^ TAC mice that received AAV9‐miR‐204 (Figure 3G,H). The collagen genes (*Col1a1*, *Col1a2* and *Col3a1*) expression analysis also showed decreased levels in the hearts of miR‐204^–/–^ TAC mice receiving AAV9‐miR‐204 (Figure S5D). A decrease in the cardiac fibrosis and collagen genes expression could be because of leaky upregulation of miR‐204 in fibroblast, the outcome of a decreased pathological change in the heart, or a combination of both. The cross‐sectional area of cardiomyocytes was significantly low in the heart of miR‐204^–/−^ TAC mice that received AAV9‐miR‐204 compared to those that received control virus (Figure S5E). These data demonstrate the in vivo cardioprotective role of miR‐204.

### 2.3 miR‐204 regulation of APJ signalling

**FIGURE 4:**
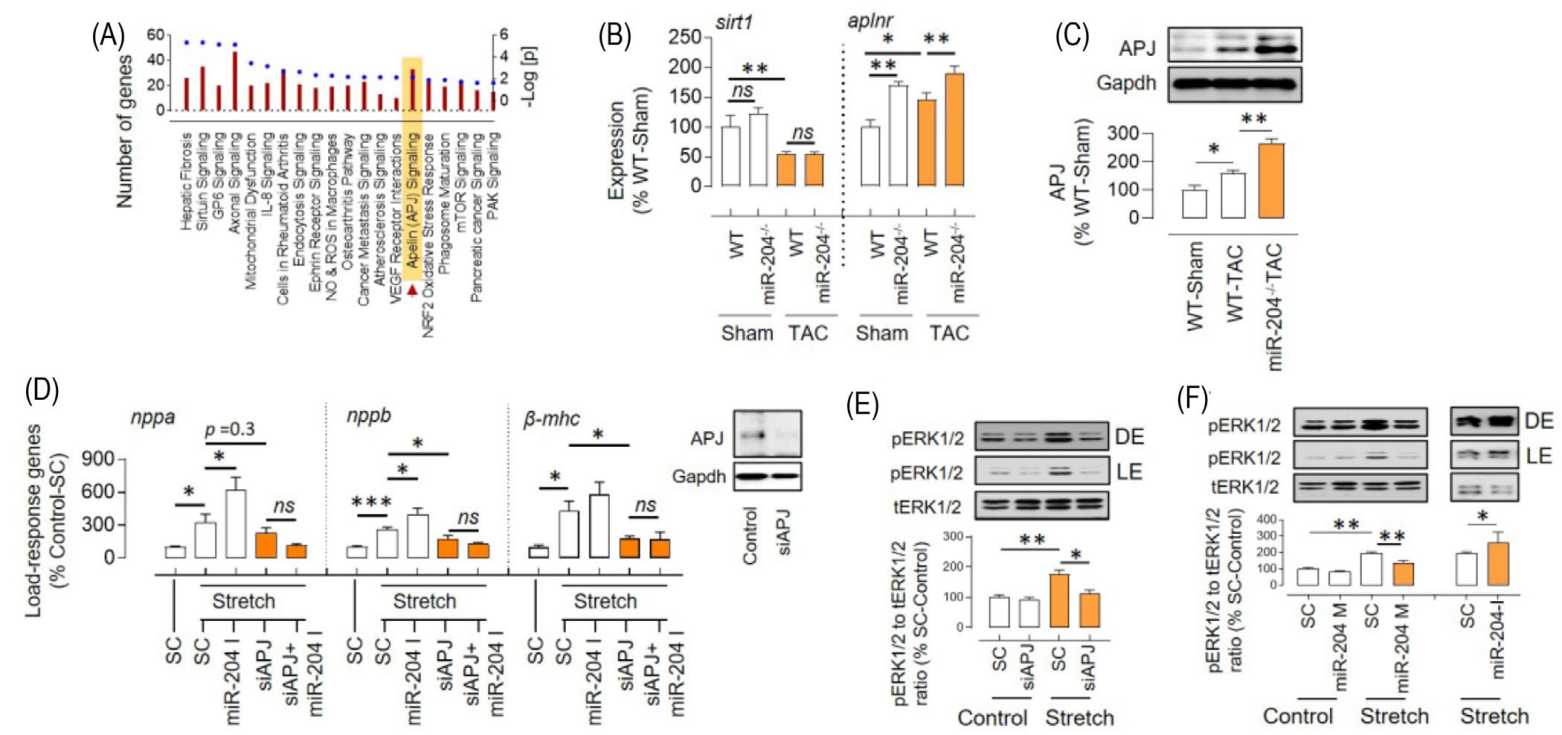
miR‐204 inversely regulates APJ‐mediated stretch response. (A) Top 20 pathways that differ in hearts of WT‐TAC versus miR‐204^–/–^ TAC mice, based on ingenuity pathway analysis of gene expression profiles. The left *y*‐axis indicates the number of genes linked with the pathway (bar), and the right *y*‐axis indicates the significance (dot) (*n* = 4). (B) The expression levels of *sirt1* and *aplnr* genes in the heart of WT and miR‐204^−/−^ sham/TAC mice (*n* = 5–15). (C) Increased levels of APJ in the heart of miR‐204^–/–^ TAC mice compared to that of WT‐TAC mice (*n* = 5). (D) Effect of APJ silencing (50 nM) and miR‐204 inhibition (100 nM) on load‐response gene expression in H9C2 cells during the stretch (*n* = 6). Insert shows effect of siRNA (50 nM) on APJ silencing. (E) The stretch‐induced ERK1/2 activation in the setting of APJ silencing (50 nM) (*n* = 3). (F) Effect of miR‐204 mimic (M, 100 nM) and inhibitor (I, 100 nM) on the stretch‐induced increase in pERK1/2 in H9C2 cells. Data are shown as mean, and error bars represent SEM (*n* = 4). *p < .05, **p < .01, versus indicated group. SC, scrambled control; pERK1/2, phosphorylated extracellular signal‐regulated kinase 1/2; tERK1/2, total extracellular signal‐regulated kinase 1/2; *nppa*, natriuretic peptide a; *nppb*, natriuretic peptide b; *β‐mhc*, beta‐myosin heavy chain; LE, light exposure; DE, dark exposure

Ingenuity pathway analysis of the transcriptomics of TAC hearts isolated from WT and miR‐204^–/–^ mice suggested Sirtun1 and APJ signalling pathways (Figure 4A, GSE185595). We validated the expression levels of *sirt1* and *aplnr* (genes encoding for sirtuin1 and APJ) in the heart of WT and miR‐204^–/−^ mice. The miR‐204^–/–^ TAC mice had significantly higher levels of *aplnr* compared to the WT‐TAC mice (Figure 4B). Besides, the miR‐204^–/–^ mice had a higher level of *aplnr* at baseline compared to the WT and miR‐204^+/–^ mice (Figure S6A). Next, we examined the expression APJ protein and found that miR‐204^–/–^ TAC mice had significantly higher levels of APJ (Figure 4C). The miRs typically have multiple targets, and miR‐204 could mediate its effects via other pathways. To determine the dependence of miR‐204's effects on APJ, we measured the effects of miR‐204 inhibition on load‐response gene induction in H9C2 cells in which APJ was silenced (>80%). The silencing of APJ not only prevented a stretch‐induced increase in load‐response genes but also blocked their upregulation by miR‐204 inhibition (Figure 4D). The phosphorylation of extracellular signal‐regulated kinase 1/2 (pERK1/2) during stretch, marks the initiation of the hypertrophic response and that depends on APJ.^24^, ^25^ Consistent with APJ and miR‐204 interaction in the regulation of hypertrophic response (*nppa*, *nppb* and *β‐mhc*) of H9C2 cells, the levels of pERK1/2 during stretch depended on APJ (Figure 4E) and was inversely regulated by miR‐204 (Figure 4F). Based on our results and the known role of APJ in heart failure,^24^, ^26^ we focused on the interplay between APJ and miR‐204 during TAC.

### 2.4 miR‐204 promotes APJ endocytosis

**FIGURE 5:**
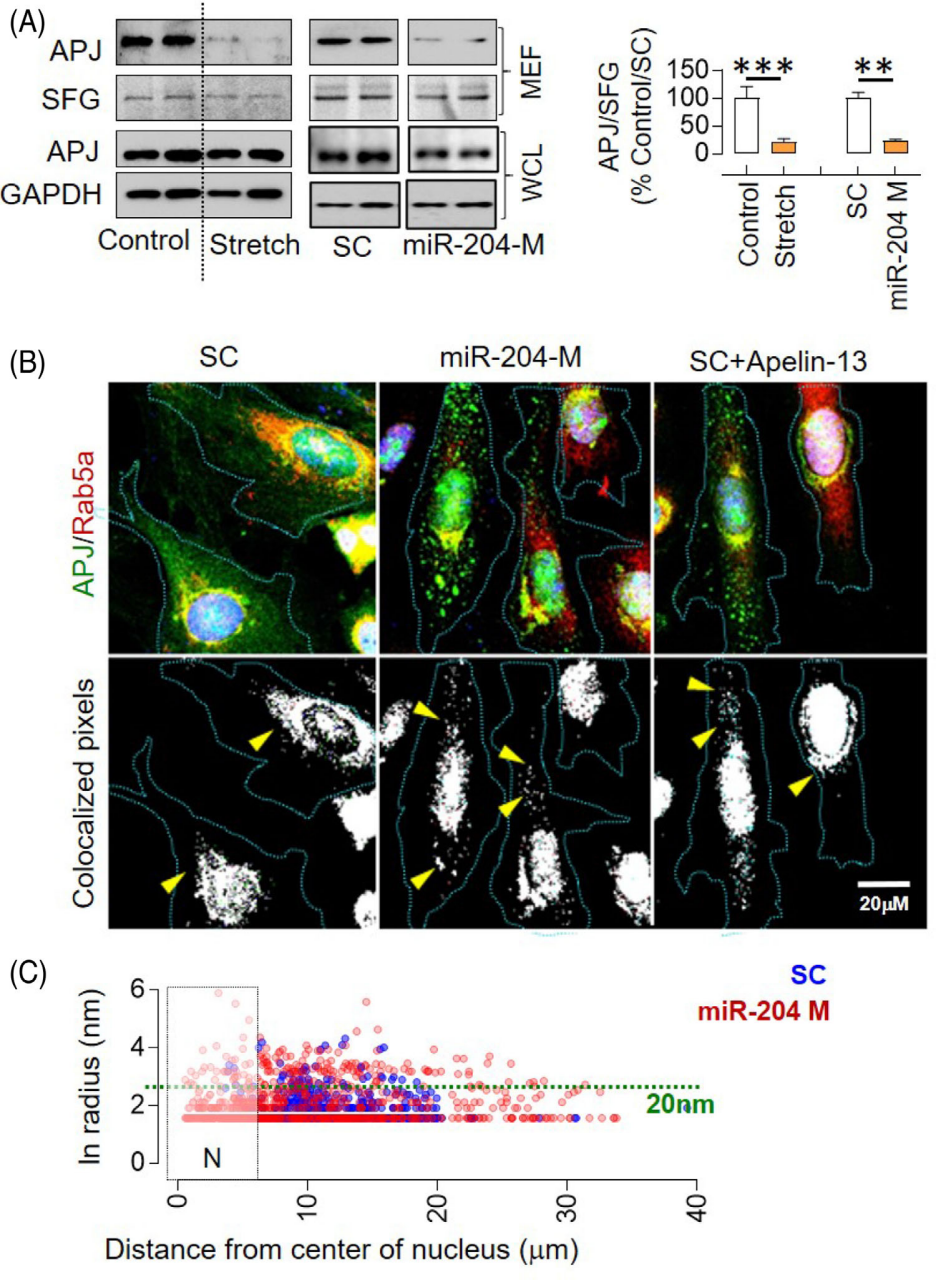
miR‐204 induces APJ endocytosis. (A) Effect of stretch and miR‐204 overexpression on the APJ levels in membrane‐enriched fraction (MEF) and whole‐cell lysate (WCL) of H9C2 cells and its quantification (*n* = 4). The immunoblot for the miR‐204 experiment (right panel) originates from the same gel, and the uncropped image is provided in Figure S12. (B) Colocalisation of APJ with Rab5a (endosomal marker) in response to miR‐204 overexpression, as determined using the RG2B ImageJ plugin (arrowheads). H9C2 cells treated with apelin‐13 (1 h, 10 nM) served as a positive control; ×63 magnification. (C) The *XY* plot shows the radius of APJ and Rab5a‐positive puncta on the *y*‐axis (nm) and its distance from the nucleus on the *x*‐axis (μm). Quantification was done by ImageJ ‘analyse particles’ plugin. ‘*N*’ marks the nuclear boundary; the green dotted line marks a 20‐nm radius (*n* = 5). Data are shown as mean, and error bars represent SEM. **p < .01, ***p < .001, versus indicated group. SC, scrambled control; miR‐204 M, miR‐204 mimic; SFG, stain‐free gel

Stretching H9C2 cells for 24 h led to a significant decrease in the expression levels of APJ in the membrane‐enriched fraction (MEF), despite no change in the whole‐cell lysate (WCL) (Figure 5A). This suggested translocation of APJ from the membrane to the cytoplasm and was not surprising as stretching activates the β‐arrestin pathway, promoting receptor desensitisation and endocytosis. Next, we investigated whether miR‐204 regulates the endocytosis of APJ in H9C2 cells. We found that miR‐204 also induced a significant decrease in APJ levels in the MEF of H9C2 cells, despite no change in the WCL (Figure 5A). Prior studies show that the APJ expression declines during culture in the primary cardiomyocytes,^24^ but we did not observe any appreciable change in the expression of *aplnr* (encoding for APJ) in the H9C2 cells cultured for four passages (Figure S6B). The time and concentration‐dependent decline in MEF APJ by miR‐204 confirmed that the miR‐204 decreases surface availability of APJ in the H9C2 cells (Figure S6C). Next, to ascertain whether miR‐204 promotes endocytosis of APJ, we measured the colocalisation of APJ with the endosomal marker, Rab5a, in H9C2 cells overexpressing miR‐204 or treated with APJ ligand, apelin‐13 (positive control). We found that miR‐204 overexpression led to the accumulation of APJ in the cytoplasm of H9C2 cells, forming a punctate structure, and approximately 50% of APJ‐positive puncta colocalised with Rab5a (Figure 5B,C). A significant proportion of APJ resided around the nucleus at the basal condition, very little colocalises with Rab5a and larger APJ puncta were rare (Figure 5B). Both miR‐204 overexpression and apelin‐13 treatment individually increased the number of APJ puncta larger than 20 nm and were located farther from the nuclear boundary (Figure 5C). Given that Rab5a only marks early endosomes,^27^ many APJ containing puncta that are perhaps in different stages did not colocalize with Rab5a.

### 2.5 miR‐204 regulates APJ endocytosis through the dynamin‐independent and Ca^2+^‐sensitive pathway

**FIGURE 6:**
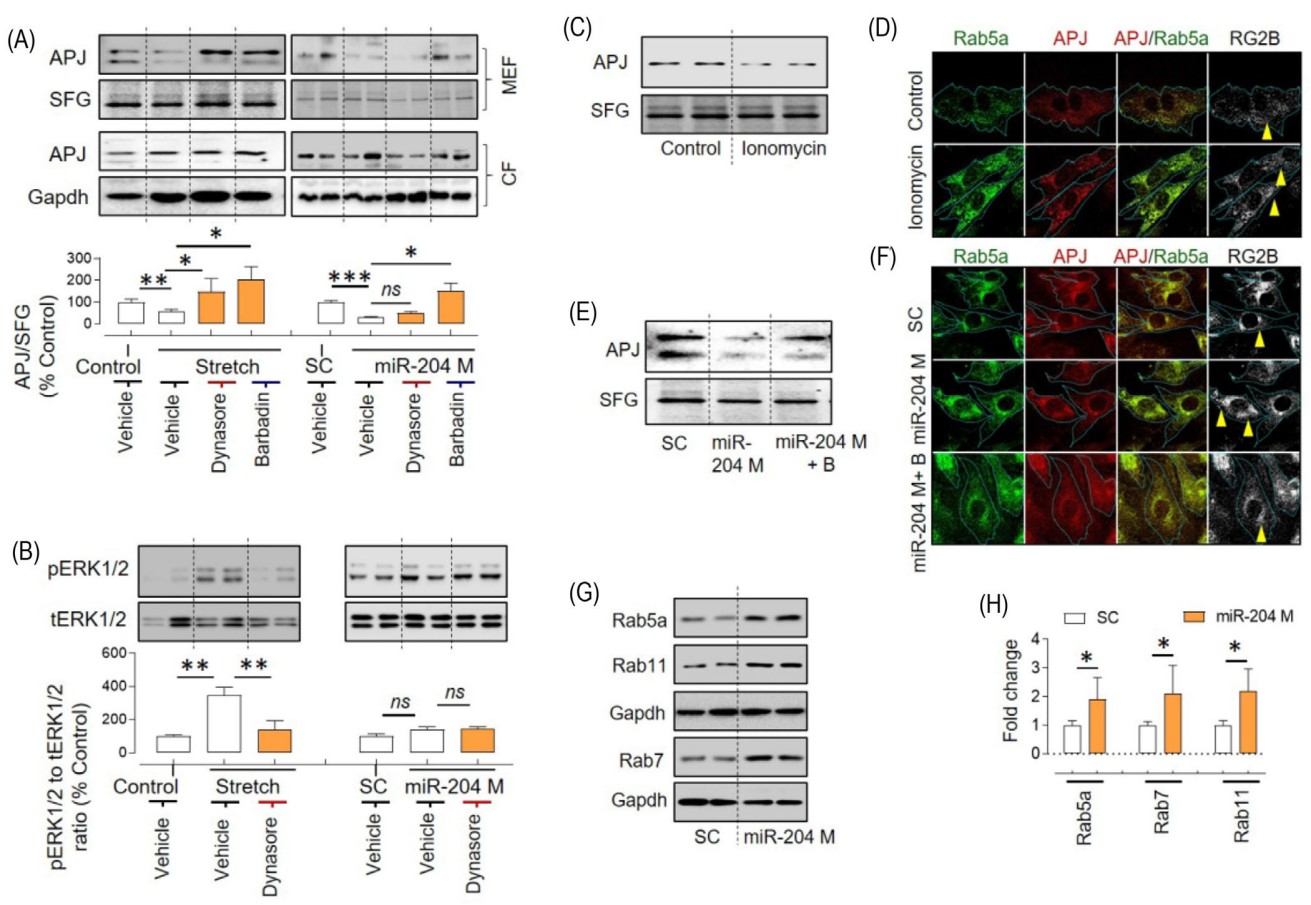
Dynamin‐independent, Ca^2+^‐sensitive regulation of APJ endocytosis by miR‐204 in H9C2 cells. (A) Effects of dynasore (100 μM) and barbadin (100 μM) on APJ expression in the MEF and WCL following stretch (24 h) or miR‐204 overexpression (24 h) in H9C2 cells. Quantification of APJ in the MEF (*n* = 5). (B) Effects of dynasore on ERK1/2 activation following stretch or miR‐204 overexpression (*n* = 5). (C) Effects of ionomycin on APJ expression in the MEF in Ca^2+^ supplemented media. (D) Representative images of cardiomyocytes showing the effect of ionomycin on the formation of Rab5a and APJ puncta and their colocalisation in Ca^2+^‐supplemented media. (E) Effects of BAPTA‐AM (B, 2 μM, 24 h) on the miR‐204‐induced decrease in APJ expression in the MEF. (F) Representative images of cardiomyocytes showing the effect of miR‐204 overexpression and BAPTA‐AM (2 μM, 24 h) on the formation of Rab5a and APJ puncta and their colocalisation. In (D) and (F), the pixel colocalisation of APJ and Rab5a was determined using the RG2B plugin of ImageJ, and images were captured at ×100 magnification. (G and H) Effects of miR‐204 overexpression on the levels of Rab5a, Rab7 and Rab11 in H9C2 cells (G) and quantification (H). *p < .05, **p < .01, ***p < .001 versus indicated group. Data are shown as mean, and error bars represent SEM. B, BAPTA‐AM; SC, scrambled control; miR‐204 M, miR‐204 mimic; SFG, stain‐free gel

G‐protein‐coupled receptor's (GPCR) endocytosis typically begins with binding to β‐arrestin and adapter protein‐2 (AP‐2), trafficking to clathrin‐coated pit, and clipping from the membrane by dynamin. This endocytosis pathway can be blocked by dynasore, which inhibits dynamin, and barbadin, which inhibits the formation of the β‐arrestin‐AP‐2 complex.^28^ Here, we used dynasore and barbadin to identify whether this pathway is being employed by stretch and miR‐204 to induce APJ endocytosis. We found that both dynasore and barbadin inhibit stretch‐induced APJ endocytosis (Figure 6A). On the other hand, miR‐204‐induced APJ endocytosis was inhibited by barbadin, but not by dynasore (Figure 6A). The dynamin‐independent endocytosis of APJ by miR‐204 was ascertained by another dynamin‐inhibitor dyngo (Figure S6D). In addition, the stretch‐induced ERK1/2 activation, which is an APJ‐dependent response (Figure 4E), was inhibited by the dynasore (Figure 6B). In contrast, the miR‐204 did not induce ERK1/2 activation despite inducing APJ endocytosis, and neither was it affected by the dynasore (Figure 6B). Dynamin‐independent endocytosis has been reported in multiple cell types.^29^, ^30^, ^31^ For example, in glial cells, dynamin‐independent Ca^2+^‐regulated endocytosis is reported.^32^ Furthermore, in nerve cell clusters, entering Ca^2+^ inhibits dynamin and arrests endocytosis.^33^ Thus, we examined the role of Ca^2+^ in APJ endocytosis using ionomycin, an antibiotic that increases intracellular Ca^2+^,^34^ and BAPTA‐AM, a Ca^2+^ chelator that decreases intracellular Ca^2+^.^35^ Ionomycin induced APJ endocytosis in H9C2 cells, whereas BAPTA‐AM inhibited miR‐204‐induced APJ endocytosis (Figure 6C–F and Figure S7A–C). We measured the intracellular Ca^2+^ 24 h after miR‐204 transfection and found a significant increase (Figure S7D). These results suggest that miR‐204‐induced increase in Ca^2+^ contributes to its effects on the APJ endocytosis.

Gene ontology analysis of miR‐204‐target genes expressed in the heart shows that miR‐204 deregulates pathways associated with endocytosis, cell–cell adhesion, transcription and microtubule anchoring to the centrosome (Figure S8). Moreover, ingenuity pathway analysis of differentially regulated genes in the hearts of miR‐204^–/–^ TAC mice also shows deregulation of the endocytosis pathway (Figure 4A). Thus, we investigated whether miR‐204 affects the expression of endocytosis mediators and found that miR‐204 upregulates the ras‐associated binding proteins (Rab5a, Rab7 and Rab11), but does not affect the expression of β‐arrestin1/2, early endosome‐associated protein (EEA1), caveolin 1 and clathrin (Figure 6G,H and Figure S9A,B). These results suggest that miR‐204 can regulate cellular endocytosis in general, and if this is the case, it could affect the endocytosis of other receptors. To test this, we measured the miR‐204's effect on α2 and β3 adrenergic receptors in the MEF of HEK293 cells. We found that miR‐204 overexpression upregulated the α2 adrenergic receptor, whereas there was no effect on the β3 adrenergic receptor (Figure S9C,D). Thus, despite miR‐204 regulating the endocytosis machinery, its effects appear to be receptor specific. Though both stretch and miR‐204 induce APJ endocytosis, the mechanism and the signalling outcome are distinct.

### 2.6 miR‐204 changes the surface availability of APJ in vivo during pressure overload

**FIGURE 7:**
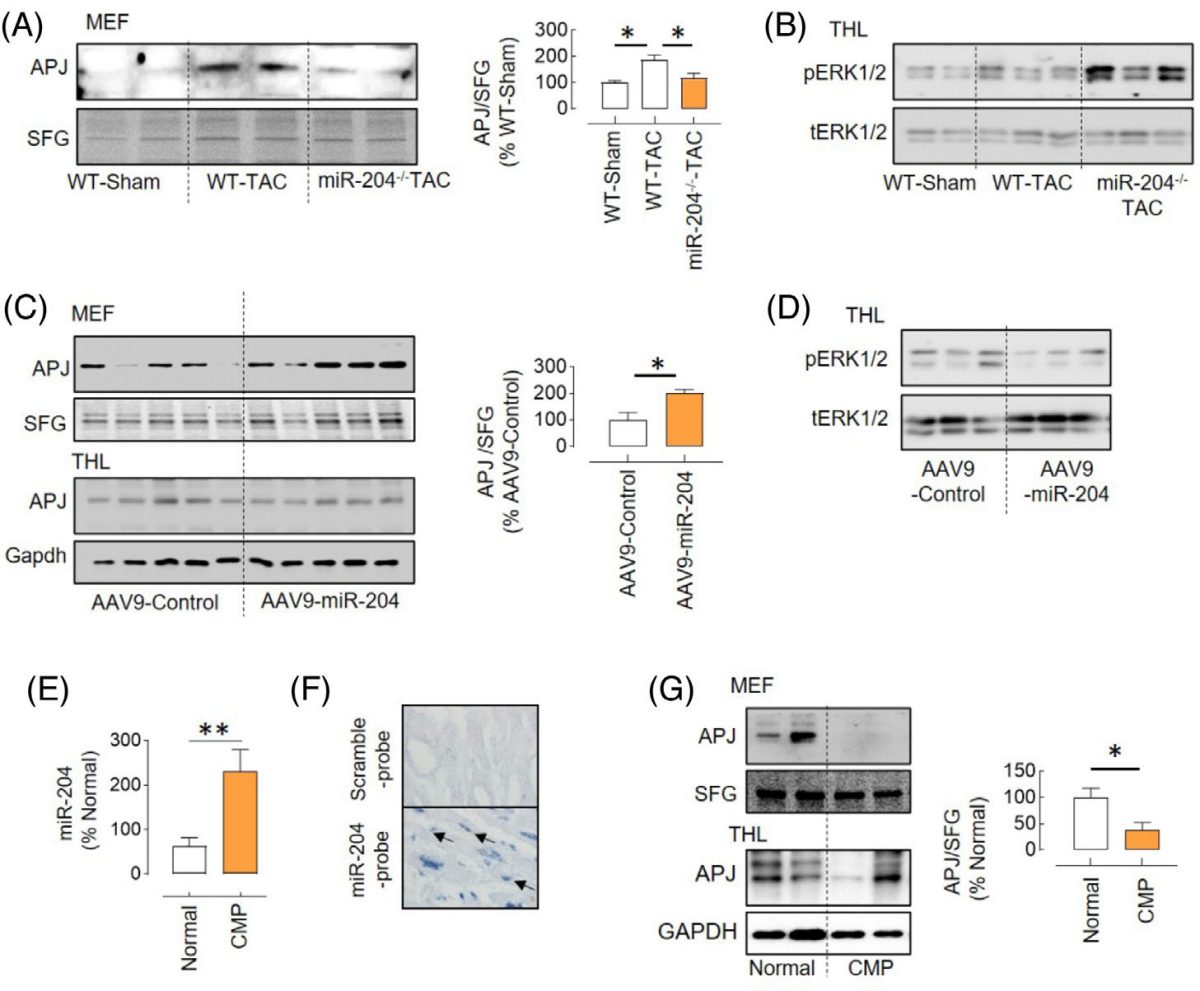
miR‐204 regulation of APJ trafficking in vivo. (A) The APJ expression in the MEF of WT‐TAC and miR‐204^–/–^ TAC mice and its quantification (*n* = 4). (B) Immunoblot showing increased levels of pERK1/2 in the heart of miR‐204^−/−^ TAC mice compared to WT‐TAC mice. (C) The APJ expression in the MEF of the heart of miR‐204^–/–^ TAC mice receiving AAV9‐miR‐204 or AAV9‐control virus and its quantification (*n* = 5). (D) Immunoblot showing decreased levels of pERK1/2 in the heart of miR‐204^–/–^ TAC mice provided with AAV9‐miR‐204 compared to those that received control virus. (E) miR‐204 expression in the heart of cardiomyopathy (CMP) patients or normal donors (*n* = 9–10). (F) In situ hybridisation showing miR‐204 (arrows) in cardiomyocytes of the cardiomyopathy patient heart. (G) The APJ expression in the MEF and total heart‐tissue lysate (THL) of normal donors and CMP patients and its quantification (*n* = 5). *p < .05, **p < .01 versus indicated group. Data are shown as mean, and error bars represent SEM. SFG, stain‐free gel; scramble‐probe, scrambled control probe

To determine the in vivo effect of miR‐204 on APJ trafficking during pressure overload, we measured APJ expression in the total heart lysate and MEF of hearts isolated from the WT‐TAC and miR‐204^–/–^ TAC mice. TAC increased the APJ expression in the total heart lysate (Figure 4C) and MEF (Figure 7A) of WT mice. Notably, despite even higher levels of APJ in the total heart lysate of miR‐204^–/−^ TAC mice (Figure 4C), its expression in the MEF was lower (Figure 7A and Figure S10A). In consistence, we found that the miR‐204^–/−^ TAC mice that were provided with AAV9‐miR‐204 had a higher APJ in the MEF, despite no change in the total APJ expression (Figure 7C). Differential regulation of endogenous APJ ligands (e.g., apelin and elabela) by miR‐204 can affect the surface availability of APJ. However, we did not find any difference in the expression of apelin (gene and protein) and elabela (gene) in the heart of WT‐TAC and miR‐204^–/−^ TAC mice (Figure S10B–D). These findings demonstrate that miR‐204 has a role in APJ trafficking in vivo during hypertrophic stress. However, in contrast to an inverse association between miR‐204 levels and APJ in MEF of H9C2 cells (Figure 5A), we observed a positive association between miR‐204 levels and APJ in MEF of the heart of TAC mice (Figure 7A,C). We also found that the miR‐204^–/–^ TAC mice have higher pERK1/2 levels, the effect which was reversed in miR‐204^–/–^ TAC mice that received AAV9‐miR‐204 virus (Figure 7B,D).

### 2.7 miR‐204 and APJ interaction in the human cardiomyopathy

We explored the significance of miR‐204 and APJ in human cardiomyopathy. We obtained ventricular tissue from explanted hearts with ventricular dysfunction as assessed by left ventricular ejection fraction from patients undergoing heart transplants.^36^ We compared the miR‐204 expression levels and APJ in these hearts to those of subjects who had no known heart disease, normal left ventricular function and died of noncardiac causes. Compared to normal human hearts, cardiomyopathic hearts showed increased levels of miR‐204 and lower levels of APJ in the MEF (Figure 7E–G).

## 3 DISCUSSION

The miR‐204 is located in the intron 6 of the *TRPM3* gene on chromosome 9q21.12. It is expressed in multiple tissues and has been shown to affect the development of vascular calcification, endothelial dysfunction, pulmonary artery hypertension, osteoarthritis and retinal disorders.^20^, ^37^, ^38^, ^39^ The published reports indicate the potential role of miR‐204 in regulating cardiac function,^5^, ^10^, ^11^, ^12^, ^13^ though the biological significance of miR‐204 in the development of CH/CD remains unknown. The upregulation of miR‐204 in the H9C2 cells by cyclic stretching and angiotensin‐II, in NRCMs by phenylephrine, in mouse hearts by pressure overload and in the human cardiomyopathic hearts shows that it is positively regulated during stress.

To test the causal role of miR‐204 in the heart, we used global miR‐204‐5p knockout mice. The miRs are expressed in pairs (3p and 5p). We find that the miR‐204^–/–^ mice had ∼40% decline in the cardiac level of miR‐204‐3p in addition to >95% decline in miR‐204‐5p (Figure S3B). The miR strand sorting depends on multiple factors, such as tissue types, development stages, disease and the abundance of target genes.^40^, ^41^, ^42^ However, whether the absence of complementary miR‐204‐5p sequence in miR‐204^–/–^ mice affects the precursor hairpin stability or arm sorting remains unknown. Considering >100‐fold lower abundance of miR‐204‐3p in the heart compared to miR‐204‐5p and only 40% decline in the miR‐204^–/–^ mice, we think that decrease in miR‐204‐5p primarily drives the observed effects. miR‐204 targets hundreds of genes and could mediate its effects via multiple pathways. Therefore, we performed an unbiased bioinformatics analysis of miR‐204^–/–^ TAC mice's cardiac transcript profile, which suggests deregulation of multiple pathways, including APJ and sirtuin1. Recently, we reported that sirtuin1, a nicotinamide adenosine dinucleotide (NAD)‐dependent deacetylase, mediates its cardiac effects via regulation of the acetylation of the sodium channel (NaV1.5).^36^ However, the validation experiments supported the deregulation of the APJ pathway in the heart of miR‐204^–/−^ TAC mice (Figure 4B). The silencing of APJ not only prevented a stretch‐induced increase in load‐response genes but also blocked their induction by miR‐204 inhibition (Figure 4D). Besides, we find that the stretch‐induced ERK1/2 activation depends on the APJ, and is inversely regulated by the miR‐204 (Figure 4E,F). Our findings showing that ERK1/2 activation by stretch depends on APJ were consistent with the previous reports.^24^, ^25^

APJ is a GPCR that senses endogenous ligands (apelin) and mechanical stretch. APJ is emerging as a molecular target for a variety of diseases, including heart failure,^43^, ^44^, ^45^, ^46^, ^47^, ^48^, ^49^ and polymorphism in APJ gene is associated with a risk for cardiovascular disorders.^50^, ^51^, ^52^ APJ signalling consists of G‐protein and β‐arrestin‐dependent pathways. The ligand‐induced G‐protein pathway mediates cardioprotective effects,^24^, ^26^, ^53^ whereas the stretch‐induced β‐arrestin pathway promotes CH/CD.^24^ During pressure overload, the β‐arrestin pathway dominates over the G‐protein pathway, as mice lacking APJ are protected from pressure overload‐induced CH/CD, whereas mice lacking apelin are not.^24^, ^48^ Conversely, exogenous apelin rescues pressure overload‐induced cardiac hypertrophy.^24^, ^26^, ^53^ Moreover, a G‐protein‐biased APJ agonist, MM07, provides protective effects in the animal model of pulmonary arterial hypertension.^54^ Therefore, a selective inhibition of the ability of APJ to respond to stretch, opposed to a general agonism, is expected to provide cardioprotective effects. In general, stretch receptors are activated either by a change in the lateral pressure profile or by extracellular anchorage to other proteins, and this requires the availability of these receptors on the cell membrane.^55^ Our in vivo results showed that the miR‐204^–/−^ mice are susceptible to CH/CD following TAC (Figure 2) and have lower surface availability of APJ despite an increase in the total APJ expression (Figures 4C and 7A), and both phenotypes are reversed by cardioselective overexpression of miR‐204 (Figures 3 and 7C). Considering that the stretch decreased the surface availability of APJ (Figure 5A) and miR‐204 rescued the stretch response in H9C2 cells (Figures 1C and 4F), we anticipated that the miR‐204 would inhibit the stretch‐induced APJ endocytosis. In contrast, we found that miR‐204 also induces APJ endocytosis, though the follow‐up experiments demonstrated that it does so via a distinct mechanism (Figure 6). Perhaps, stretch‐induced APJ endocytosis is required to mediate the stretch response, while miR‐204 circumvents this via alternative APJ endocytosis.

Endocytosis of GPCRs is a critical mechanism that ensures their functionality.^56^, ^57^ β2 adrenergic and the M3 acetyl‐choline muscarinic receptor traffic constitutively using a clathrin‐independent mechanism.^58^ However, in the presence of an agonist, they switch to a clathrin‐dependent mechanism.^58^ The GPCRs, depending on the stimulus, can undergo a distinct endocytosis‐recycling pathway involving endosomes with an alternate signalling environment (e.g., G‐proteins, Ras, Rac and Src).^59^, ^60^, ^61^ A single GPCR can mediate distinct signalling outcomes as a function of their cellular location, such as cell membrane, endosomes, and Golgi complex.^62^, ^63^ Moreover, upon activation, the GPCRs are known to form an endosomal β‐arrestin/GPCR complex. Such complex could serve as a signalling scaffold for the ERK1/2.^64^, ^65^, ^66^ Depending on the ligand (apelin‐36 or apelin‐13), the APJ forms a stable or unstable complex with β‐arrestins, respectively.^67^ Therefore, a stable endosomal complex of APJ and β‐arrestin formation during a stretch is the potential mechanism for the ERK1/2 activation, though this needs to be experimentally verified. Previously, miR‐133α and miR‐199a/b were shown to affect the recycling of GPCR (neurotensin receptor 1)^68^, ^69^ and the expression of endocytosis mediators.^70^ We show that miR‐204 decreased surface expression of APJ and induced formation of APJ puncta (>20 nm) in the cytoplasm, which colocalised with the endosomal marker Rab5a (Figure 5). We observed a decrease in the APJ levels in the MEF but not in the WCL, suggesting that the effect of miR‐204 on APJ is not mediated via APJ gene silencing (Figure 5A). Although we did not observe any appreciable change in APJ levels in H9C2 cells during culture (Figure S6B), a decline in APJ expression in primary cardiomyocytes 24 h post isolation is reported during culture,^24^ which could be attributed to an immediate change in the environment. However, this observation also raises the possibility that the miR‐204‐dependent mitigation of stress indirectly activates the endocytosis mechanisms leading to decreased expression of APJ on the cell surface. The bioinformatic analysis of the 3' untranslated region of the APJ gene shows that it does not have a conserved binding site for the miR‐204‐5p. Therefore, the miR‐204 does not affect APJ expression but rather APJ localisation via regulating the expression of endocytosis mediators (e.g., Rab5a, Rab7, Rab11). We further show that miR‐204‐induced APJ endocytosis is dependent on the intracellular Ca^2+^ (Figure 6E,F). Recently, it was reported that miR‐204 stimulates T‐type Ca^2+^ channels in isolated rat ventricular cardiomyocytes.^71^ We postulate a model that the endosomes employed by miR‐204 for APJ endocytosis are not associated with ERK1/2 activation while that employed by stretch does. Our observations in H9C2 cells showing that stretch‐induced ERK1/2 activation is inhibited by endocytosis inhibitors (Figure 6A), miR‐204 inhibits stretch‐induced ERK1/2 activation despite promoting APJ endocytosis (Figure 4F), and miR‐204^–/−^ TAC mice have higher levels of pERK1/2, which is decreased by the cardioselective miR‐204 overexpression (Figure 7B,D) supports this model.

Both static^72^ or cyclic^73^ stretch induces hypertrophic changes in the cardiomyocytes; the cyclic stretch mimics volume overload.^74^ As pressure overload and volume overload induce differential cardiac hypertrophy and signalling pathways,^75^ a further exploration of miR‐204's role during a static stretch is needed. In the present study, we collected data from the global miR‐204^–/−^ mice, and to ascertain the role of cardiac miR‐204, these findings need to be validated in the cardiomyocyte‐specific miR‐204^–/–^ mice. APJ mediates both G‐protein and β‐arrestin‐mediated signalling. APJ is needed for the apelin to induce a contractile response in the cardiomyocytes.^24^ Based on our in vitro finding showing that miR‐204 induces APJ endocytosis, we expect that miR‐204 will render APJ unavailable for both G‐protein and β‐arrestin signalling. Our in vivo data show that miR‐204 overexpression increases surface availability of APJ, and in such conditions, we expect mice to be more sensitive to the cardioprotective effects of G‐protein‐biased APJ ligands (e.g., MM07). Therefore, the functional delineation of miR‐204 and APJ interaction using APJ and miR‐204 double knockout mice and assessment of beneficial effects MM07 are interesting approaches and should be explored in future studies. We recognize the discrepancy of APJ surface availability by miR‐204 in H9C2 cells (Figure 6) compared to that in mice (Figure 7), which could be because of compensatory APJ regulation and the disease stage. In pressure overload subjected hearts, we observed a compensatory increase in the miR‐204 levels at 2 and 5 weeks after TAC surgery (Figure S3F), but whether it declines later and aggravates cardiac dysfunction and whether that can be rescued by miR‐204 intervention remains unknown. The increase in miR‐204 in the heart of cardiomyopathy patients and the functional influence of miR‐204 in CH/CD support the clinical relevance of our findings. However, the extent to which the effects of miR‐204 rely on APJ remains underexplored and warrants further studies in mice lacking APJ. Moreover, a lower APJ in the MEF of the heart of cardiomyopathy patients, despite significantly higher ventricular miR‐204, could be due to the stage of heart failure. Additional experiments in the hearts with milder dysfunction are needed to ascertain the biological relevance of miR‐204/APJ‐trafficking in human heart failure.

**FIGURE 8:**
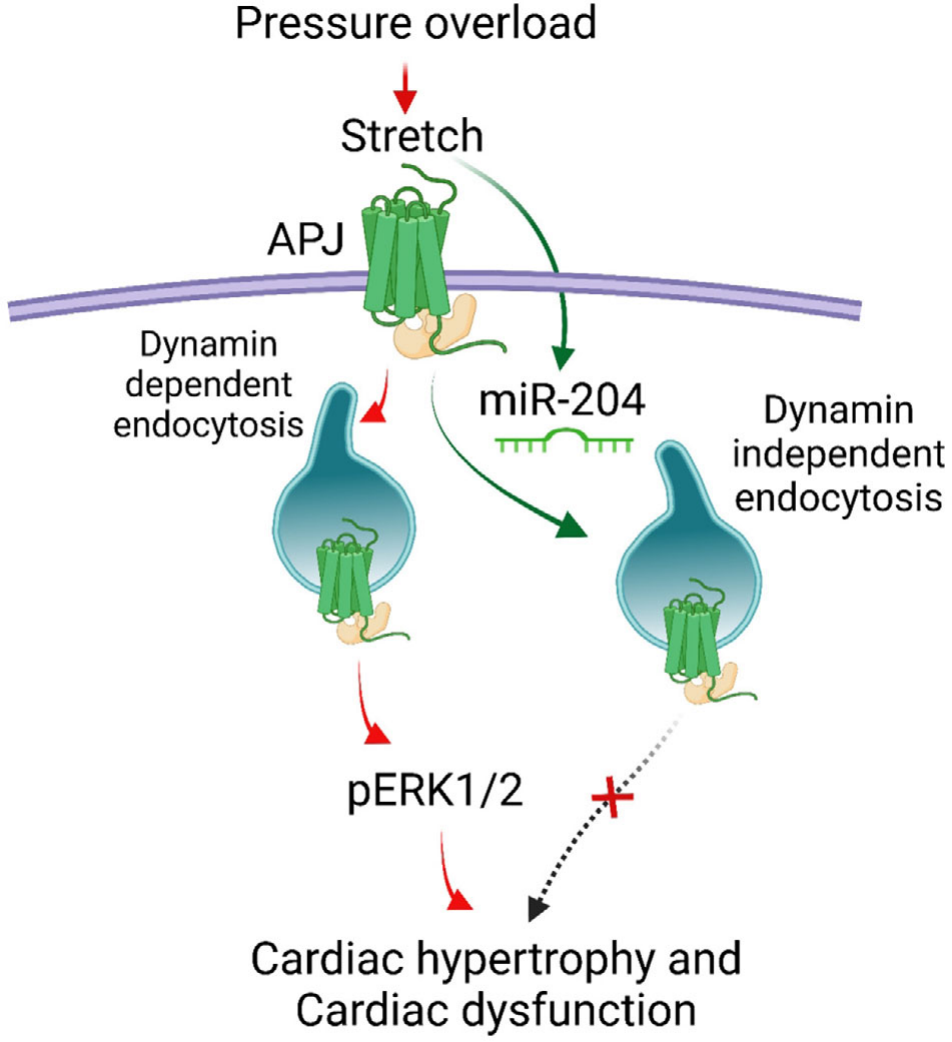
Schematic shows that the mechanical stretch stimulates APJ signalling during pressure overload, which involves dynamin‐dependent receptor endocytosis and activation of extracellular receptor kinase 1/2 (pERK1/2), leading to a hypertrophic response (red arrows). Stretch stimulates miR‐204 expression. miR‐204 suppresses APJ‐mediated stretch signalling and confers cardioprotection by triggering a shift to an alternate dynamin‐independent APJ endocytosis (green arrow) that neither activates pERK1/2 and nor promotes hypertrophic response of stretch

The heart physiologically responds to mechanical stretch at the cellular level, and prolonged stretching of cardiomyocytes during human diseases (e.g., aortic stenosis and arterial hypertension) leads to heart failure. The increase in cardiac strain, cardiac hypertrophy, and fibrosis progressively increases the risk of heart failure. We find that the stretch and miR‐204 both promote endocytosis of APJ but through a distinct mechanism. Besides, unlike stretch, the miR‐204‐induced APJ endocytosis did not lead to a hypertrophic response in the H9C2 cells. Therefore, we think that stress stimulates miR‐204, and miR‐204 promotes alternate endocytosis of APJ, leading to impairment in the ability of APJ to mediate maladaptive stretch signalling. Our results demonstrate that mice lacking miR‐204 were susceptible to pressure overload‐induced cardiac hypertrophy and dysfunction, and the miR‐204 overexpression rescues this phenotype. We also find that the upregulation of cardiac miR‐204 is a compensatory cardioprotective mechanism, which inhibits the ability of APJ to mediate maladaptive signalling and dampens the cardiac response to hypertrophic stress. Thus, we think that miR‐204 is an attractive research target for myocardial hypertrophy and heart failure. Perhaps, in the hypertrophic phase of cardiac remodelling, miR‐204 tonically regulates the hypertrophic response, and boosting miR‐204 in the heart could be beneficial (Figure 8). Our results demonstrate the suitability of developing therapeutic strategies for heart failure based on cardioselective miR‐204 intervention.

## 4 MATERIALS AND METHODS

### 4.1 Mice

All the studies were performed in C57BL/6 male WT, miR‐204^+/–^, and miR‐204^–/–^ mice, and these mice were maintained in specific pathogen‐free conditions at the central animal facility of the University of Iowa, IA. The baseline cardiac parameters were assessed in both male and female mice. The objective of this study was to determine the role of miR‐204 in the development of CH/CD. In mice with either a heterozygous (miR‐204^+/–^) or homozygous (miR‐204^–/–^) deletion of miR‐204, the thoracic aorta was surgically constricted to 0.4 mm, creating a phenotype that mimics pressure overload. At 5 weeks, the mice were terminated, we used the heart to perform the morphometric, histological and molecular analyses. Next, we used adeno‐associated virus 9 (AAV9) to overexpress miR‐204 in the heart of miR‐204^–/–^ mice cardioselectively and to determine whether this can reverse the effects of pressure overload. All experimental groups included randomly chosen littermates, about 8 weeks old and from the same strain. All animal experiments were approved by the Institutional Animal Care and Use Committee (IACUC) of the University of Iowa and were carried out according to the National Institute of Health (NIH) guidelines.

### 4.2 Cells

Neonatal rat cardiomyocytes (NRCMs) were isolated from newly delivered neonatal Sprague–Dawley rat pups using the neonatal cardiomyocyte isolation system (Worthington Biochemical Co, Lakewood, NJ). H9C2, NRCMs and HEK293 cells were cultured in Dulbecco's modified Eagle's medium (DMEM) supplemented with 10% (v/v) fetal bovine serum and antibiotics (100 U/ml each of penicillin and streptomycin) at 37°C in humidified air with 5% CO_2_. The H9C2 cells were exposed to 500 nM of angiotensin II, whereas NRCMs were exposed to 10 μM of phenylephrine for 24 h to induce cellular hypertrophy.

### 4.3 Trans‐aortic constriction

The pressure overload phenotype in mice was induced by TAC, as described.^76^ Briefly, mice were anesthetised with inhalational isoflurane (2%–3%), intubated orally with a 20‐gauge tube, and ventilated with a small rodent ventilator (Harvard Apparatus, Holliston, MA). Respiratory rate and body temperature were monitored continuously during the procedure. A thoracotomy was created between the second and third intercostal space, and the aortic arch was visualised. Aortic constriction was performed by tying a 7‐0 Prolene suture ligature against a 27‐gauge needle, yielding a 0.4‐mm diameter when the needle was removed. In sham mice, the aortic arch was visualised but not constricted.

### 4.4 Echocardiography

Transthoracic echocardiograms were performed in the Cardiology Animal Phenotyping Core Laboratory at the University of Iowa, using a Vevo 2100 Imager (VisualSonics, Toronto, ON, Canada), as described previously.^77^ Briefly, conscious sedation was achieved with midazolam (0.2–0.3 mg; subcutaneous). The anterior chest was shaved, and the prewarmed ultrasonic gel was applied. Two‐dimensional (2D) images were acquired in the left ventricle (LV) short‐ and long‐axes planes, with a 40‐MHz linear array probe, yielding 200 frames per second. The biplane area‐length method was used to calculate the LV mass, thickness, volumes, ejection fraction (EF) and fractional shortening (FS). The longitudinal and radial strains were determined by speckle tracking analysis using VevoStrain Software (Visual Sonics, Toronto, ON, Canada).

### 4.5 AAV9 virus injections

A single injection of AAV9 control (AAV2/9 CMV‐IRES eGFP) and AAV9‐miR‐204 (AAV2/9 CMVmiR‐204‐IRES eGFP) virus (100 μl containing 5 × 10^11^ viral genomes) was performed on anesthetised miR‐204^–/–^ mice 1 week before TAC surgery. Briefly, mice were anesthetised, and a small incision was made through the skin to expose the jugular vein. The AAV9 control and AAV9‐miR‐204 virus were injected into the jugular vein. A tiny droplet of vet bond was used to stop bleeding, and the incision was sutured with a nonabsorbable suture.

### 4.6 RNA isolation and real‐time polymerase chain reaction (RT‐PCR)

RNA was isolated using Qiazol/Trizol, as per the manufacturer's instructions. miRs were converted to cDNA using the qscriptTM microRNA cDNA synthesis kit (Quanta Biosciences, Gaithersburg, MD, USA). RT‐qPCR for miR‐204 (mature and precursor), *nppa, nppb*, *β‐mhc*, *Col1a1*, *Col1a2*, *Col3a1, Sirt1, Apln, Apela* and *Aplnr* was performed using the Brilliant II SYBR Green RT‐qPCR kit. 18S was used as an internal control. The primer sequences are provided in Table S3.

### 4.7 RNA sequencing

The RNA was quantified using a fluorimetric RiboGreen assay, and RNA integrity was assessed using capillary electrophoresis and generating an RNA integrity number (RIN) (Agilent BioAnalyzer 2100). The samples with higher than 500 ng and RIN of 8 or greater were used to generate Illumina sequencing libraries using Illumina's TruSeq RNA Sample Preparation Kit (Cat. # RS‐122‐2001 or RS‐122‐2002) or Stranded mRNA Sample Preparation Kit (Cat. # RS‐122‐2101). The libraries were amplified using 15 cycles of PCR, and the final library size distribution was validated using capillary electrophoresis and quantified using fluorimetry (PicoGreen) and via Q‐PCR. Indexed libraries are then normalised, pooled and size‐selected to 320 bp (tight) using the PippinHT instrument. Pooled libraries are denatured and diluted to the appropriate clustering concentration. The libraries are then loaded onto the NovaSeq paired‐end flow cell, and clustering occurs onboard the instrument. Once clustering is complete, the sequencing reaction immediately begins using Illumina's two‐colour SBS chemistry. Upon completion of reading 1, a 7‐base pair index read is performed in the case of single‐indexed libraries. If dual indexing was used during library preparation, two separate 8‐ or 10‐base pair index reads are performed. Finally, the clustered library fragments are resynthesised in the reverse direction, thus producing the template for paired‐end read 2. Base call files for each cycle of sequencing are generated by Illumina Real‐Time Analysis (RTA) software. The base call files and run folders are streamed to servers maintained at the Minnesota Supercomputing Institute. Primary analysis and de‐multiplexing are performed using Illumina's bcl2fastq v2.20.

### 4.8 Transfection of oligonucleotides

Cells (H9C2, NRCMS and HEK293) were transfected with oligonucleotides using Lipofectamine 2000 (Invitrogen). Oligos (scrambled control, miR‐204 mimic and miR‐204 inhibitor) were incubated with Lipofectamine 2000 (3 μl) at room temperature for 20 min and then added to the cells in basal DMEM (3 ml) supplemented with antibiotics. The cells were kept in the transfection mixture for 24–32 h at 37°C in humidified air with 5% CO_2_. The sequences of oligonucleotides used in the study are provided in Table S3.

### 4.9 Stretching experiments

The H9C2 cells were cultured on a flexible‐bottomed six‐well plate coated with laminin (Flexcell International, Burlington, NC) in DMEM (supplemented with 10% (v/v) fetal bovine serum and antibiotics) 48 h before mechanical stretch. Cells were subjected to biaxial cyclic stretch produced by a Flexcell FX‐2000 strain unit (Flexcell International) with a computer‐controlled application of sinusoidal negative pressure (20%) by a vacuum at a frequency of 0.5 Hz (30 cycles/min) for 24 h. For transfection experiments, cells were transfected either with miR‐204 mimic or inhibitor in serum‐free DMEM 8 h before starting mechanical stretch. Cells were collected 24 h after stretch and stored at −80°C until further processing.

### 4.10 Membrane‐enriched fraction isolation

The WCL was prepared and separated into an MEF as previously described.^78^ Briefly, after homogenisation in a buffer containing 0.1% NP‐40, the MEF was isolated by serial centrifugation. The efficiency of separating the MEF was determined by measuring the expression level of ATPase (Na‐K) α1 (Figure S11). Expression of APJ in the WCL was normalised to Gapdh, whereas expression in the MEF was normalised to the stain‐free gel (SFG).

### 4.11 Immunoblotting

Protein samples were resolved on 10%–12% SDS‐PAGE and transferred to nitrocellulose membranes. MEF protein samples were resolved on Criterion TGX precast gels (4%–20%). Antigen–primary antibody complexes were incubated with horseradish‐peroxidase (HRP)‐conjugated secondary antibodies and visualised by Western blotting luminol reagent (Thermo USA). Anti‐APJ (Millipore‐Sigma #ABD43), anti‐apelin (LSbio‐Rabbit mAb #LS‐C149244‐200), anti‐Rab5a (Cell Signaling‐Mouse mAb #46449), anti‐caveolin‐1 (Cell Signaling‐Rabbit mAb #3267), anti‐clathrin‐heavy chain (Cell Signaling‐Rabbit mAb #4796), anti‐EEA‐1 (Cell Signaling‐Rabbit mAb #3288), anti‐Rab7 (Cell Signaling‐Rabbit mAb #9367), anti‐Rab‐11 (Cell Signaling‐Rabbit mAb #5589), anti‐pERK1/2 (Cell Signaling‐Rabbit mAb #9101), anti‐tERK1/2 (Cell Signaling, mAb #9102), anti‐α2a adrenergic receptor (Alomone‐Rabbit mAb#AAR‐020), anti‐β3 adrenergic receptor (Abcam‐Rabbit mAb#ab94506), anti‐caspase‐3 (Cell Signaling, mAb #9662), anti‐ATPase (Na‐K) α1 (DSHB‐A6F‐C) and anti‐GAPDH (R&D Systems‐Rabbit mAb #2275‐PC‐020) were used at a working dilution of 1:1000. Images were captured and quantified using Image Lab (BioRad, USA) software, and intensity values were normalised to Gapdh. For the MEF, the protein band in the stain‐free gel image was used for the normalisation. The uncropped blots are included in the Figures S12 and S13.

### 4.12 Immunofluorescence staining

H9C2 cells were fixed by 4% paraformaldehyde, permeabilised using 0.1% Triton‐X and incubated with primary antibodies. For the immunofluorescence experiments, the working concentration of anti‐APJ (Millipore‐Sigma# ABD43) and anti‐Rab5a (Cell Signaling‐Mouse mAb #46449) antibodies was 1:100. Antigen‐primary antibody complexes were probed with fluorescence‐tagged secondary antibodies, and images were captured using a Zeiss confocal microscope (Model 710 and 510). The nucleus was counterstained with DAPI. Cardiomyocytes treated with apelin were used as the positive control; cardiomyocytes treated with scrambled control served as a negative control. Pixel colocalisation of APJ with Rab5a was determined using the ImageJ software colocalisation plugin, RG2B.^36^ The confocal images were colour separated to generate an image that showed only the APJ signal. Then the green channel image (showing APJ) and the red channel image (showing Rab5a) were processed to identify pixel colocalisation of APJ and Rab5a. The distance and distribution of APJ/Rab5a puncta in both images were determined using the particle analysis function of ImageJ. The distance between individual puncta was calculated from the centre of the nucleus. We performed this analysis in six cardiomyocytes from each experimental condition, measuring the size and distance of >1000 puncta from each cell. The data are plotted as an *XY* distribution, where the *X*‐axis represents the distance of the particle from the nucleus, and the *Y*‐axis represents the size of the puncta.

### 4.13 Cell size measurement

The cell size was determined as described with some modifications.^79^, ^80^ Briefly, the cells were fixed in 4% paraformaldehyde, followed by staining with either rhodamine‐conjugated wheat‐germ agglutinin (WGA; Life Technologies; 1:200 dilution) or actin (SC‐8432, Santa Cruz Biotechnology). The images were captured using a Zeiss confocal microscope (Model 710) and Image J was used to measure the area of the cells.

### 4.14 Tissue staining

Myocardial tissues were fixed in a 4% formalin solution. The cardiac sections (6 μm) were stained using Sirius Red. ImageJ software quantified the percentage of fibrosis (brown) in Sirius Red staining. We used five fields from each animal for the analysis of fibrosis. The cardiac sections were stained with rhodamine‐conjugated WGA (Life Technologies; 1:200 dilution) for cell border detection and DAPI for nuclei detection for cross‐sectional area measurement. Images from areas of transversely cut cardiac slices were taken using confocal microscopy (×20 magnification) at 633 nm for WGA. The cross‐sectional area of cardiomyocytes was measured using ImageJ software.

### 4.15 In silico analysis

MicroRNA‐SVR is an algorithm that ranks microRNA–mRNA target site interaction by assigning an SVR score. A lower SVR score indicates a stronger interaction between a microRNA and an mRNA target site.^81^ First, miR‐204 target genes were tabulated at different binding strengths (threshold SVR score < −0.9 and < −0.3; www.microRNA.org, data available as tab‐delimited files). Then, based on RNA sequencing of the heart, transcripts representing <0.0002% of the total transcripts were excluded from the analysis. Next, we prepared two lists of genes: (a) those that are expressed in the heart and had an SVR score of <−0.9, and (b) those that are expressed in the heart and had an SVR score of <−0.3. The first list included 319 genes, whereas the second list included 1100 genes. Gene‐ontology analysis was performed using the Database for Annotation, Visualization, and Integrated Discovery (DAVID) v6.8 from the Laboratory of Human Retrovirology and Immunoinformatics (LHRI). The biological process was analysed, and we identified the top 30 deregulated pathways that were generated based on the 319 genes. We also looked for the deregulation of these pathways in the less‐stringent analysis that yielded 1100 genes.

### 4.16 Statistical analysis

Statistical analysis was performed using GraphPad Prism (Version 8.0). One‐way analysis of variance (ANOVA) was used for multiple comparisons, and Tukey's test was used for post hoc analysis. An independent sample *t*‐test was used to determine the significance of the difference between the two groups. The data are shown as the mean, and the error bars represent the standard error of the mean (SEM). The results were considered significant where *p* < .05.

## CONFLICT OF INTEREST

The authors declare that there is no conflict of interest.

## Supporting information


**FIGURE S1** (A) The effect of 10%, 15% and 20% stretching (after 24 h) on miR‐204 expression in H9C2 cells (*n* = 3–7). (B) The effect of 20% stretching on miR‐204 expression after 1 and 24 h (*n* = 3–7). (C and D) Representative images showing the effect of stretching on the size of WGA‐stained H9C2 cells (C) and quantification (D). The area of 100 cells was measured using ImageJ. Magnification ×60; scale bar: 20 mm. (E) Effect of stretching on the caspase‐3 expression (*n* = 3). (F) Immunoblot showing adenoviral‐mediated overexpression of dominant‐negative STAT3 (Ad‐DNSTAT3) in H9C2 cells. (G) The DNSTAT3 upregulates miR‐204 expression in the H9C2 cells (*n* = 6). (H) The DNSTAT3 overexpression stimulates stretch‐induced miR‐204 upregulation in the H9C2 cells (*n* = 5). (I and J) The effect of miR‐204 inhibitor (miR‐204 I; 20 nM)) on the miR‐204 (I) and load response gene (*nppa* and *nppb*) (J) levels in the phenylephrine (PE, 10 μM, 24 h)‐treated NRCMs (*n* = 4–7). (K and L) Representative image showing the effect of miR‐204 I and PE treatment on the size of NRCMs (H), and its quantification (I). Magnification ×60; scale bar: 20 μm. The NRCMs were immunostained with actin (green) and counterstained with DAPI (red). Data are shown as the mean, and error bars represent SEM. ^ns^ *p* > .05, *p < .05, **p < .01, ***p < .001 versus indicated group. ns, not significant; SC, scrambled control; *nppa*, natriuretic peptide a*; nppb*, natriuretic peptide b; WGA, wheat‐germ agglutinin
**FIGURE S2** (A) Effect of angiotensin II (500 nM, 24 h) on the expression of miR‐204 and load‐response genes in H9C2 cells (*n* = 3). (B and C) Effect of miR‐204 mimic (B) and miR‐204 inhibitor (C) on the expression of miR‐204 in the H9C2 cells in the presence and absence of stretch (n = 5–10). Data are shown as mean, and error bars represent SEM. *p < .05, **p < .01, ***p < .001 versus indicated group. *nppa*, natriuretic peptide a*; nppb*, natriuretic peptide b; *β‐mhc*, beta‐myosin heavy chain
**FIGURE S3** Increased cardiomyocyte size and cardiac fibrosis in miR‐204^−/−^ TAC mice. (A) Genotyping of WT, miR‐204^+/−^, and miR‐204^−/−^ mice. The PCR product is 300 bp, and the restriction site for the FokI enzyme is in the 10‐bp deletion zone for the miR‐204. Therefore, the PCR product of WT mice undergo digestion, resulting in a single band at ∼150 bp (lanes 5 and 8), miR‐204^+/−^ (heterozygous) mice have two bands, 300 and 150 bp (lanes 2,3,4, 7 and 9), and miR‐204^−/−^ (homozygous) mice have a single band at ∼300 bp (lane 6). The ladder is included in lane 1. (B) The expression of miR‐204‐5p and miR‐204‐3p in the heart of the WT and miR‐204^−/−^ mice (*n* = 6). (C–E) The LV thickness (C), heart rate (D) and %EF (E) of WT, miR‐204^+/−^ and miR‐204^−/−^ male and female mice at baseline (*n* = 3–6). (F) The expression of miR‐204 in the heart of WT mice at 2 and 5 weeks after TAC surgery (*n* = 3–10). (G) Expression of fibrosis‐associated collagen genes (*Col1a1, Col1a2* and *Col3a1*) in the heart of WT‐TAC and miR‐204^−/−^ TAC mice (*n* = 4–8). (H) Wheat‐germ agglutinin (WGA) staining of myocardial sections (top) marking the membrane of cardiomyocytes. WGA staining is shown as ‘cyan’; DAPI staining of the nucleus is shown in red. The longest diameter of the cardiomyocyte was used to measure the cross‐sectional area (bottom). *n*(*N*) = 9(3)–12(4). For 'E', the replicates are shown as ‘*n*(*N*)’, where ‘*n*’ represents the number of fields and ‘*N*’ represents the number of mice. Data are shown as mean, and error bars represent SEM. *p < .05, **p < .01, ***p < .001 versus indicated group
**FIGURE S4** Effect of TAC on miR‐204 expression (A, *n* = 6–7), left ventricular weight (LVW) normalised to tibia length (TL) (B, *n* = 4–11), and percent ejection fraction (%EF) (C, *n* = 4–11) in WT and miR‐204^+/−^ (het) mice. ns, not significant. Data are shown as mean, and error bars represent SEM
**FIGURE S5** AAV9‐miR‐204 cardioselectively upregulates miR‐204 and decreases fibrosis‐associated collagen gene expression in the heart following TAC. (A) Cardioselective upregulation of miR‐204 in WT mice following a single injection of AAV9‐miR‐204 (5 × 10^11^ viral genomes per mouse) through the jugular vein (*n* = 4). (B) The schematic shows the experimental design for the in vivo administration of AAV9‐control and AAV9‐miR‐204 virus. (C) eGFP (green) immunostaining of the cardiac section of the mice that received no virus (−Ve control), AA9‐control virus and AAV9‐miR‐204 virus. Both miR‐204 encoding and control AAV9 virus contained eGFP and was expressed in the heart following intravenous administration. Magnification ×40. (D) The expression of fibrosis‐associated collagen genes (*Col1a1, Col1a2* and *Col3a1*) in the heart of miR‐204^−/−^ TAC mice that received AAV9‐miR‐204 or AAV9‐control virus (*n* = 4–5). (E) WGA staining of myocardial sections (top, magnification ×60) marking the membrane of cardiomyocytes. The longest diameter of the cardiomyocyte was used to measure the cross‐sectional area (bottom). *n*(*N*)=13(3)–15(3). ‘*n*(*N*)’ where ‘*n*’ represents the number of fields and ‘*N*’ represents the number of mice. Data are shown as mean, and error bars represent SEM. *p < .05, ***p < .001 versus indicated group
**FIGURE S6** (A) Expression of Aplnr (encoding for APJ) in the heart of WT, miR‐204^+/−^ and miR‐204^−/−^ mice (*n* = 3–9). (B) Expression of Aplnr in the H9C2 cells at passage 10 (P10) and passage 14 (P14) (*n* = 3). (C) Time and concentration‐dependent effect of miR‐204 on the APJ expression in the MEF of H9C2 cells (*n* = 3–4). (D) Effects of dyngo (1 μM) on the APJ expression in the MEF following miR‐204 overexpression (100 nM, 24 h) in H9C2 cells. Quantification of APJ in the MEF (*n* = 3–5). Data are shown as mean, and error bars represent SEM. ns, not significant; *p < .05, **p < .01, ***p < .001 versus indicated group
**FIGURE S7** Role of Ca^2+^ in the APJ endocytosis. (A) Ionomycin increases intracellular Ca^2+^ and decreases APJ expression in the MEF of cardiomyocytes (*n* = 4–5). (B) The cell‐permeable Ca^2+^ chelator, BAPTA‐AM, prevents miR‐204 mimic‐induced decrease in APJ expression in the MEF of cardiomyocytes (*n* = 3–5). (C) The *Z*‐stack of cardiomyocytes showing the three‐dimensional effect of miR‐204 mimic and BAPTA‐AM on Rab5a (green) and APJ (red) colocalisation. The *x*, *y* and *z* axes are indicated. In control (SC) cardiomyocytes, APJ‐positive signal is localised near the nucleus. miR‐204 mimic‐treated cardiomyocytes have the formation of Rab5a‐positive puncta, which colocalise with the APJ puncta. The addition of BAPTA‐AM decreases the miR‐204 mimic‐induced Rab5a puncta formation and its colocalisation with APJ in the cardiomyocytes. (D) The quantification and representative image for Ca^2+^ (Fluo‐3 AM) in H9C2 cells 24 h after transfection with either SC or miR‐204 mimic. Magnification ×20. Data are shown as mean, and error bars represent SEM. *p < .05, **p < .01 versus indicated group. SC, scrambled control; miR‐204 M, miR‐204 mimic; B, BAPTA‐AM
**FIGURE S8** The gene‐ontology analysis of miR‐204 target genes expressed in the heart. The gene‐ontology enrichment analysis using the Database for Annotation, Visualization, and Integrated Discovery (DAVID) v6.8 shows the top 30 biological processes changed by the genes expressed in the heart and targeted by the miR‐204. (A) Includes miR‐204‐target genes with SVR score ≤ −0.3 (1100 genes). (B) Includes miR‐204‐target genes with SVR score ≤ −0.9 (319 genes)
**FIGURE S9** Effect of miR‐204 on the expression of β‐arrestins, EEA1, caveolin‐1 and clathrin. (A) The effect of miR‐204 mimic on the expression of endocytosis mediators (β‐arrestins, EEA1, Cav1 and clathrin) in H9C2 cells. (B) Quantification of proteins in ‘A’ (*n* = 4–5). (C) Immunoblots showing the effect of miR‐204 mimic on the membrane expression of the α2a‐adrenergic receptor (α2a‐ADR) and β3‐adrenergic receptor (β3‐ADR) in HEK293 cells. (D) Quantification of membrane α2‐ADR and β3‐ADR expression in ‘C’ (*n* = 6). ns, not significant; *p < .05 versus indicated group. SC, scrambled control; miR‐204 M, miR‐204 mimic; EEA1, early endosome‐associated protein 1; Cav1, caveolin 1; α2a‐ADR, alpha2a adrenergic receptor; β3‐ADR, beta3‐adrenergic receptor
**FIGURE S10** (A) Representative image showing the WGA and APJ expression in the cardiac sections of wildtype‐TAC and miR‐204^−/−^ TAC mice. Colocalisation of APJ with WGA was determined using the RG2B ImageJ plugin (magnification ×63). (B and C) The expression of *Apln* (gene encoding apelin) and *Apela* (gene encoding elabela) in the heart of WT‐Sham, WT‐TAC and miR‐204^−/−^ TAC mice. (D) The immunoblot shows the expression of apelin in the heart of WT‐Sham, WT‐TAC and miR‐204^−/−^ TAC mice and its quantification (*n* = 4). Data are shown as mean, and error bars represent SEM. ns, not significant; THL, total heart‐tissue lysate
**FIGURE S11** Purity of membrane‐enriched fraction (MEF). The measurement of ATPase (Na‐K) α1 expression in the whole‐cell lysate (WCL), MEF and a cytoplasmic fraction (CF) of H9C2 cells
**FIGURE S12** Uncropped blot for the main figures
**FIGURE S13** Uncropped blot for the supporting figures
**TABLE S1** Echocardiographic parameters of WT and miR‐204^−/−^ mice
**TABLE S2** Echocardiographic parameters of miR‐204^−/−^ TAC mice receiving AAV9‐control or AAV9‐miR‐204 virus
**TABLE S3** List of primers and their sequences

Video S1

Video S2

Video S3

Video S4
