# Supplementary material for: The microRNA‐204‐5p inhibits APJ signalling and confers resistance to cardiac hypertrophy and dysfunction
Source: Clin Transl Med. 2022 Jan 21;12(1):e693. doi: 10.1002/ctm2.693 (PMC8777385; doi:10.1002/ctm2.693)
Supplement: Supplementary file 1 — FIGURE S1 (A) The effect of 10%, 15% and 20% stretching (after 24 h) on miR‐204 expression in H9C2 cells (n = 3–7). (B) The effect of 20% stretching on miR‐204 expression after 1 and 24 h (n = 3–7). (C and D) Representative images showing the effect of stretching on the size of WGA‐stained H9C2 cells (C) and quantification (D). The area of 100 cells was measured using ImageJ. Magnification ×60; scale bar: 20 mm. (E) Effect of stretching on the caspase‐3 expression (n = 3). (F) Immunoblot showing adenoviral‐mediated overexpression of dominant‐negative STAT3 (Ad‐DNSTAT3) in H9C2 cells. (G) The DNSTAT3 upregulates miR‐204 expression in the H9C2 cells (n = 6). (H) The DNSTAT3 overexpression stimulates stretch‐induced miR‐204 upregulation in the H9C2 cells (n = 5). (I and J) The effect of miR‐204 inhibitor (miR‐204 I; 20 nM)) on the miR‐204 (I) and load response gene (nppa and nppb) (J) levels in the phenylephrine (PE, 10 μM, 24 h)‐treated NRCMs (n = 4–7). (K and L) Representative image showing the effect of miR‐204 I and PE treatment on the size of NRCMs (H), and its quantification (I). Magnification ×60; scale bar: 20 μm. The NRCMs were immunostained with actin (green) and counterstained with DAPI (red). Data are shown as the mean, and error bars represent SEM. ns p > .05, *p < .05, **p < .01, ***p < .001 versus indicated group. ns, not significant; SC, scrambled control; nppa, natriuretic peptide a; nppb, natriuretic peptide b; WGA, wheat‐germ agglutinin FIGURE S2 (A) Effect of angiotensin II (500 nM, 24 h) on the expression of miR‐204 and load‐response genes in H9C2 cells (n = 3). (B and C) Effect of miR‐204 mimic (B) and miR‐204 inhibitor (C) on the expression of miR‐204 in the H9C2 cells in the presence and absence of stretch (n = 5–10). Data are shown as mean, and error bars represent SEM. *p < .05, **p < .01, ***p < .001 versus indicated group. nppa, natriuretic peptide a; nppb, natriuretic peptide b; β‐mhc, beta‐myosin heavy chain FIGURE S3 Increased cardiomyocy [file CTM2-12-e693-s003.docx]

**
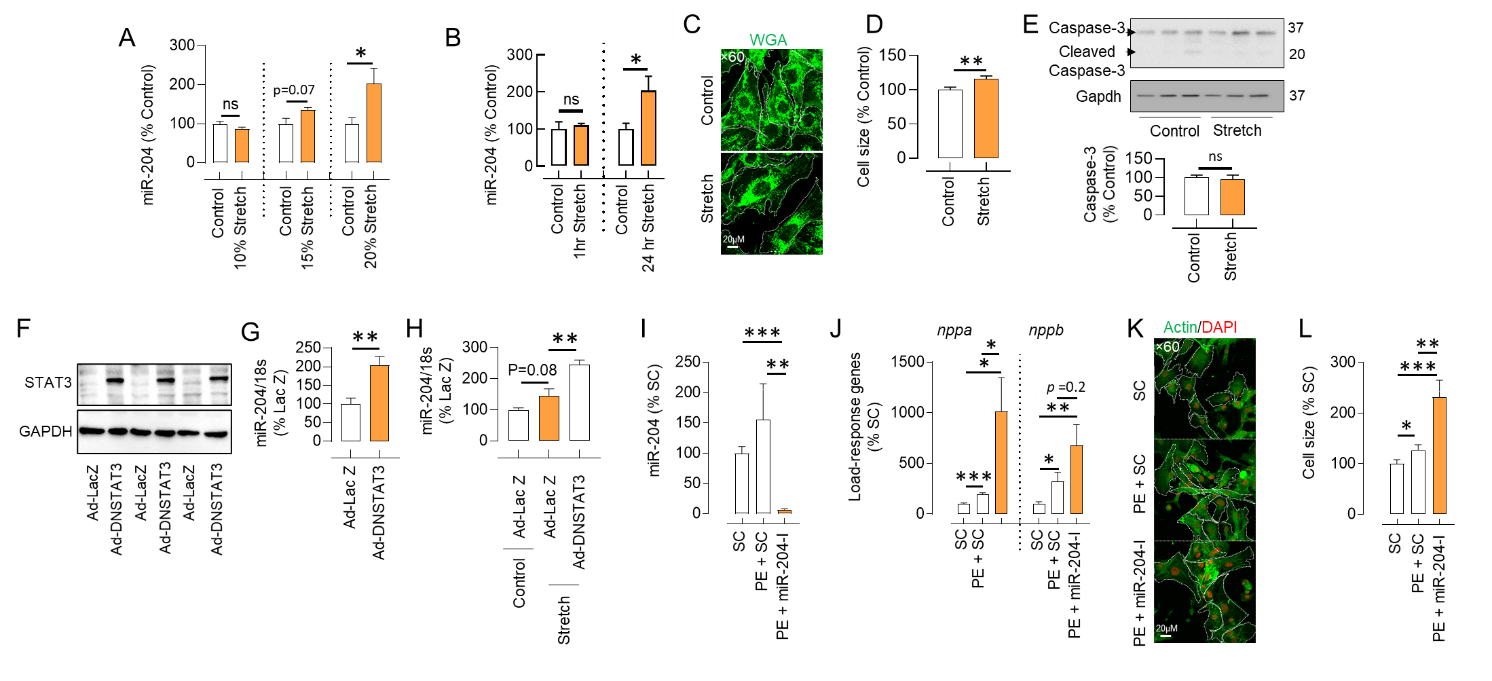
**

**Figure S1. A)** The effect of 10%, 15%, and 20% stretching (after 24 hours) on miR-204 expression in H9C2 cells. n = 3-7**. B)** The effect of 20% stretching on miR-204 expression after 1 hour and 24 hours. n = 3-7. **C & D**) Representative images showing the effect of stretching on the size of WGA stained H9C2 cells (C) and quantification (D). The area of 100 cells was measured using ImageJ. Magnification ×60, Scale bar; 20mm. **E**) Effect of stretching on the caspase-3 expression. n = 3. **F**) Immunoblot showing adenoviral-mediated overexpression of dominant-negative STAT3 (Ad-DNSTAT3) in H9C2 cells. **G**) The DNSTAT3 upregulates miR-204 expression in the H9C2 cells. n = 6. **H**) The DNSTAT3 overexpression stimulates stretch-induced miR-204 upregulation in the H9C2 cells. n=5. **I & J**) The effect of miR-204 inhibitor (miR-204 I; 20 nM)) on the miR-204 (I) and load response genes (*nppa* & *nppb*) (J) levels in the phenylephrine (PE, 10 µM, 24 hrs)-treated NRCMs. n = 4-7. **K & L**) Representative image showing the effect of miR-204 I and PE treatment on the size of NRCMs (H), and its quantification (I). Magnification ×60, scale bar; 20 µm. The NRCMs were immunostained with actin (green) and counterstained with DAPI (red). Data are shown as the mean, and error bars represent s.e.m. ^ns^p>0.05, *p < 0.05, **p < 0.01, ***p < 0.001 vs. indicated group. ns; not significant, SC; scrambled control, *nppa*; natriuretic peptide a*, nppb*; natriuretic peptide b, WGA; wheat-germ agglutinin.

**
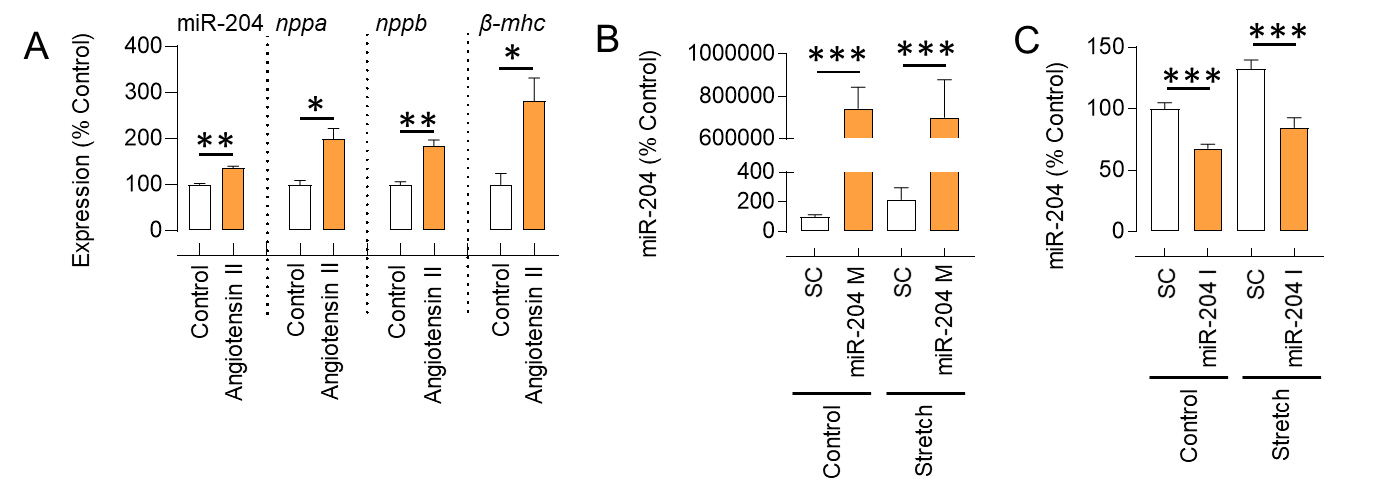
**

**Figure S2. A**) Effect of angiotensin II (500 nM, 24 hours) on the expression of miR-204 and load-response genes in H9C2 cells. n=3. **B & C**) Effect of miR-204 mimic (B) and miR-204 inhibitor (C) on the expression of miR-204 in the H9C2 cells in the presence and absence of stretch. n = 5-10. Data are shown as mean, and error bars represent s.e.m. *p < 0.05, **p < 0.01, ***p < 0.001 vs. indicated group. *nppa*; natriuretic peptide a*, nppb*; natriuretic peptide b, *β-mhc,* beta-myosin heavy chain.


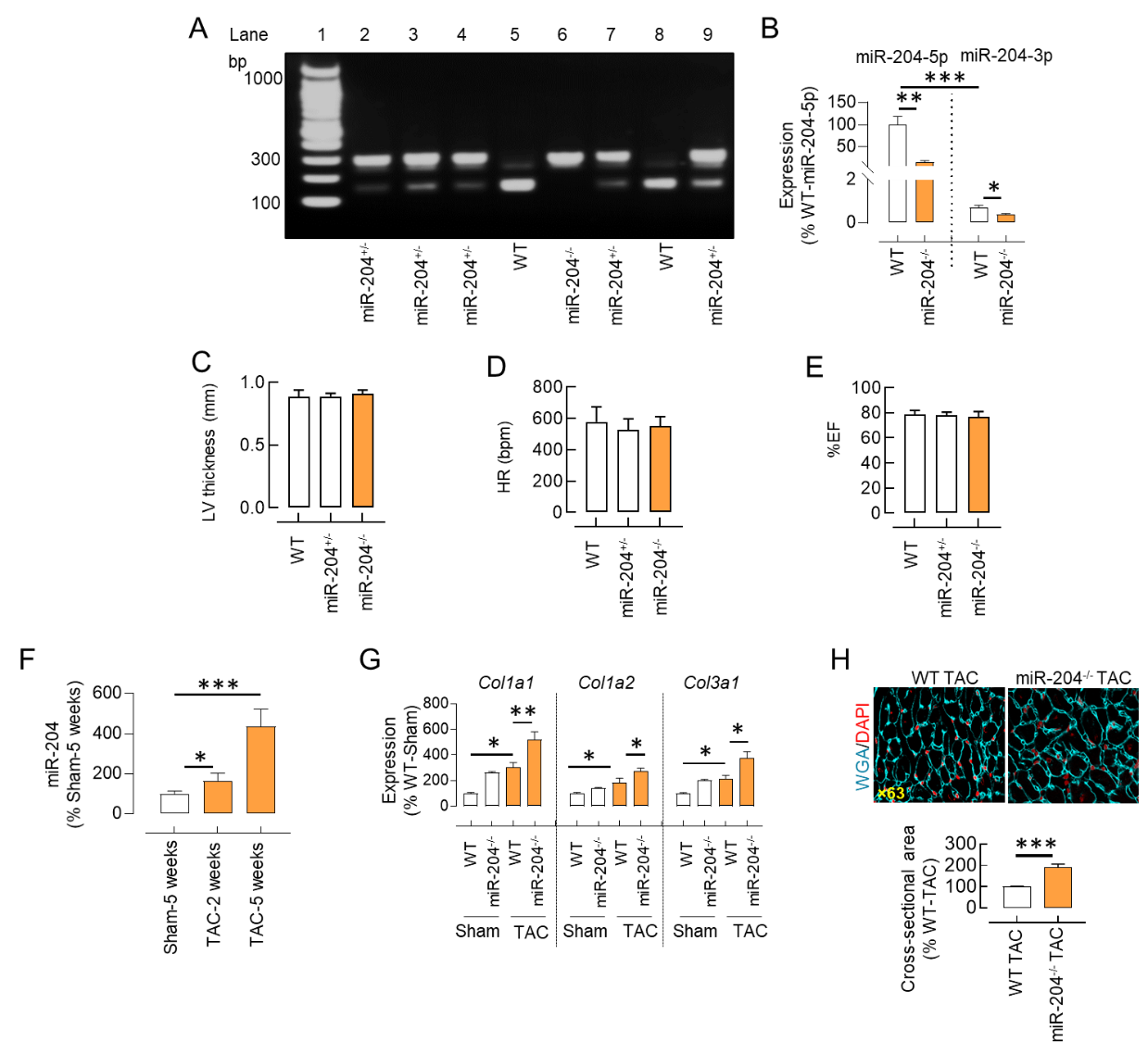


**Figure S3. Increased cardiomyocyte size and cardiac fibrosis in miR-204^-/-^ TAC mice.** **A)** Genotyping of WT, miR-204^+/-^, and miR-204^-/-^ mice. The PCR product is 300 bp, and the restriction site for the FokI enzyme is in the 10bp deletion zone for the miR-204. Therefore, the PCR product of WT mice undergo digestion, resulting in a single band at ~150 bp (lane 5 & 8), miR-204^+/-^ (heterozygous) mice have two bands – 300 and 150 bp (lane 2,3,4, 7, and 9), and miR-204^-/-^ (homozygous) mice have a single band at ~300 bp (lane 6). The ladder is included in lane 1. **B**) The expression of miR-204-5p and miR-204-3p in the heart of the WT and miR-204^-/-^ mice. n = 6. **C-E)** The LV thickness (C), heart rate (D), and % EF (E) of WT, miR-204^+/-^, and miR-204^-/-^ male and female mice at baseline. n = 3-6. **F**) The expression of miR-204 in the heart of WT mice at 2 and 5 weeks after TAC surgery. n = 3-10. **G)** Expression of fibrosis-associated collagen genes (*Col1a1, Col1a2,* and *Col3a1*) in the heart of WT-TAC and miR-204^-/-^ TAC mice. n=4-8. **H)** Wheat-germ agglutinin (WGA) staining of myocardial sections (top) marking the membrane of cardiomyocytes. WGA staining is shown as "cyan;" DAPI staining of the nucleus is shown in red. The longest diameter of the cardiomyocyte was used to measure the cross-sectional area (bottom). n(N)=9(3)-12(4). For 'E', the replicates are shown as "n(N)," where "n" represents the number of fields and "N" represents the number of mice. Data are shown as mean, and error bars represent s.e.m. *p < 0.05, **p < 0.01, ***p < 0.001 vs. indicated group.

**
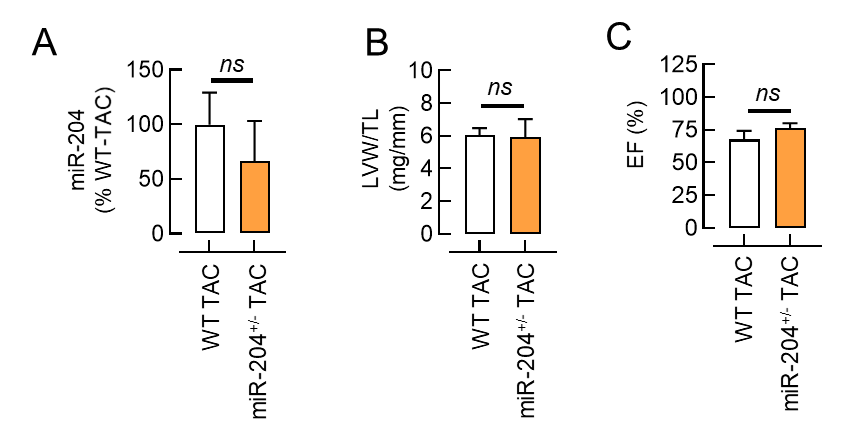
**

**Figure S4.** Effect of TAC on miR-204 expression (A, n = 6-7), left ventricular weight (LVW) normalized to tibia length (TL) (B, n = 4-11), and % ejection fraction (%EF) (C, n = 4-11) in WT and miR-204^+/-^ (het) mice. ns: not significant. Data are shown as mean, and error bars represent s.e.m.


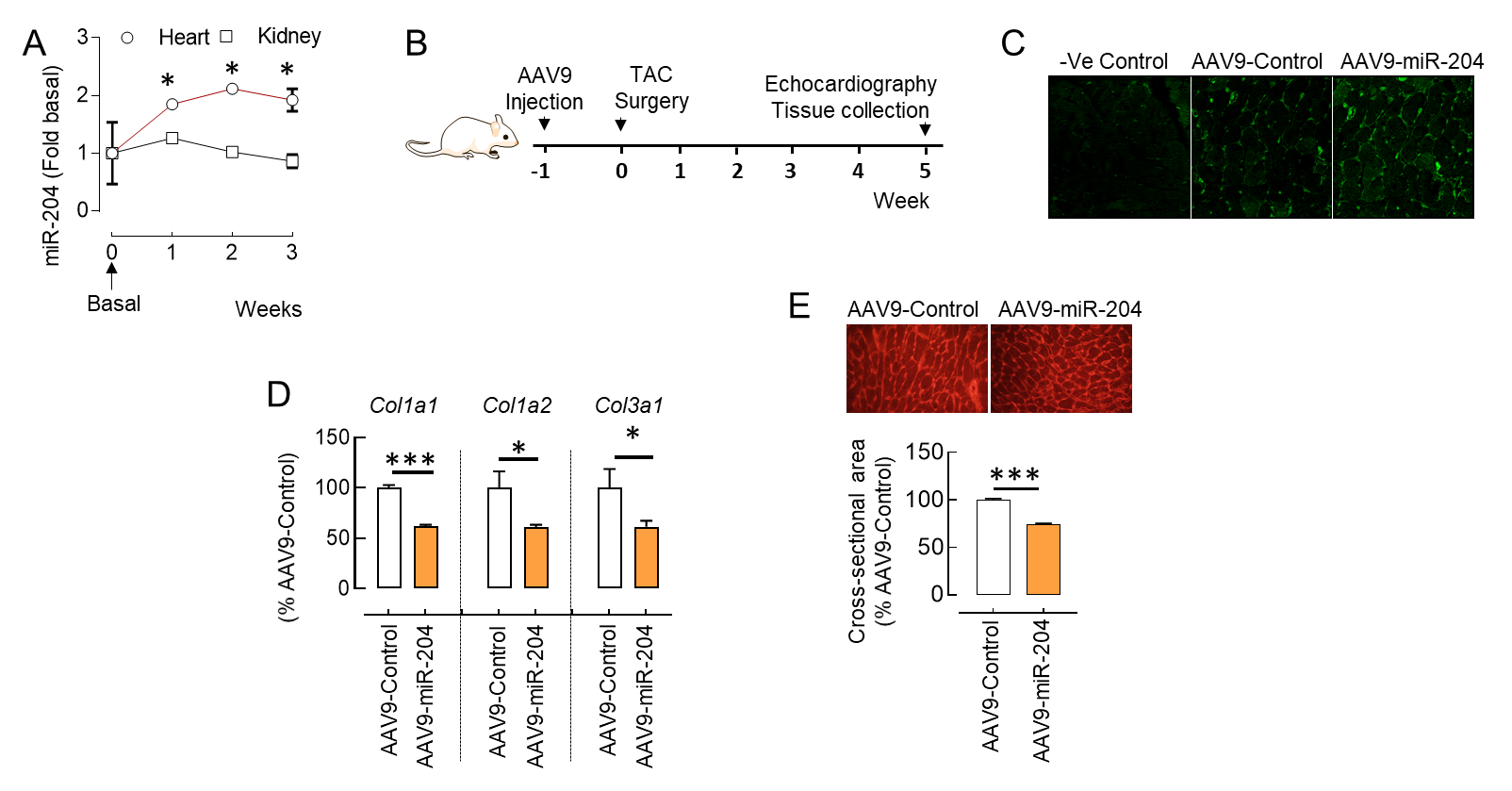


**Figure S5. AAV9-miR-204 cardioselectively upregulates miR-204 and decreases fibrosis-associated collagen gene expression in the heart following TAC. A)** Cardioselective upregulation of miR-204 in WT mice following a single injection of AAV9-miR-204 (5× 10^11^ viral genomes per mouse) through the jugular vein. n= 4. **B)** The schematic shows the experimental design for the in vivo administration of AAV9-control and AAV9-miR-204 virus. **C)** eGFP (green) immunostaining of the cardiac section of the mice that received no virus (-Ve Control), AA9-Control virus, and AAV9-miR-204 virus. Both miR-204 encoding and control AAV9 virus contained eGFP and was expressed in the heart following intravenous administration. Magnification ×40. **D)** The expression of fibrosis-associated collagen genes (*Col1a1, Col1a2,* and *Col3a1*) in the heart of miR-204^-/-^ TAC mice that received AAV9-miR-204 or AAV9-control virus. n= 4-5. **E)** WGA staining of myocardial sections (top, magnification x60) marking the membrane of cardiomyocytes. The longest diameter of the cardiomyocyte was used to measure the cross-sectional area (bottom). n(N)=13(3)-15(3). "n(N)" where "n" represents the number of fields and "N" represents the number of mice. Data are shown as mean, and error bars represent s.e.m. *p < 0.05, ***p < 0.001 vs. indicated group.


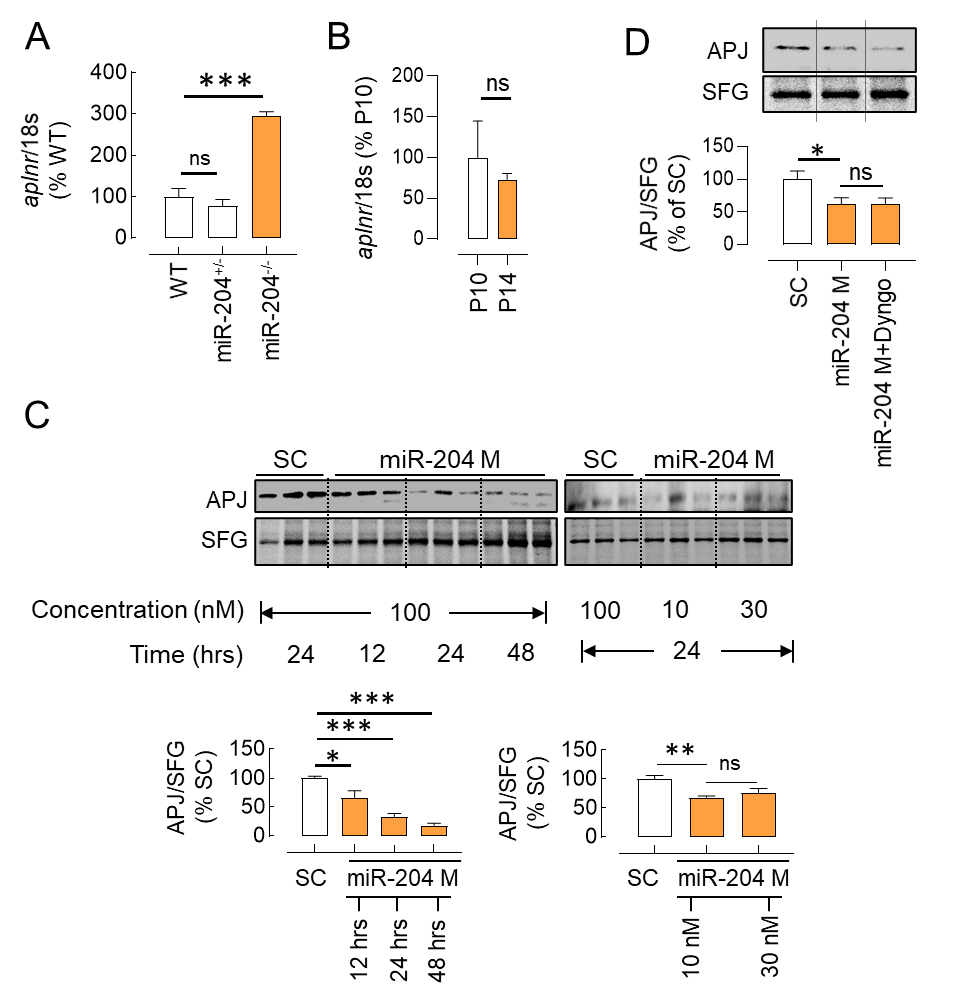


**Figure S6. A)** Expression of Aplnr (encoding for APJ) in the heart of WT, miR-204+/-, and miR-204-/- mice. n = 3-9. **B)** Expression of Aplnr in the H9C2 cells at passage 10 (P10) and passage 14 (P14). n = 3. **C)** Time and concentration-dependent effect of miR-204 on the APJ expression in the MEF of H9C2 cells. n = 3-4. **D)** Effects of dyngo (1 µM) on the APJ expression in the MEF following miR-204 overexpression (100 nM, 24 hours) in H9C2 cells. Quantification of APJ in the MEF. n = 3-5. Data are shown as mean, and error bars represent s.e.m. ns; not significant, *p < 0.05, **p < 0.01, ***p < 0.001 vs. indicated group.


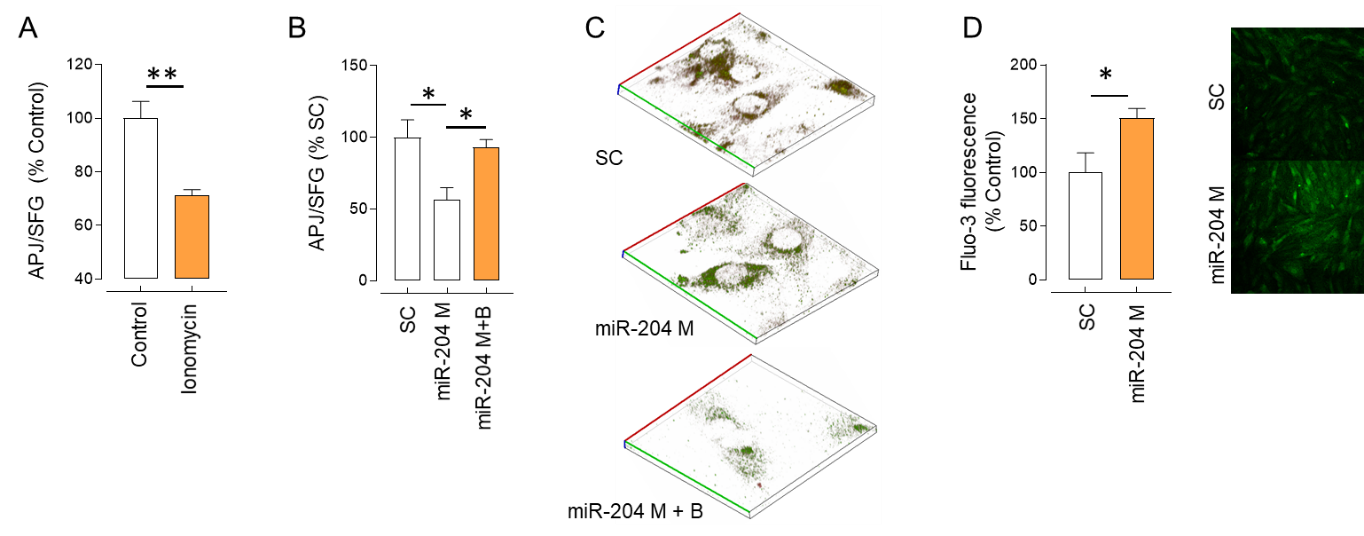


**Figure S7. Role of Ca^2+^ in the APJ endocytosis. A)** Ionomycin increases intracellular Ca^2+^ and decreases APJ expression in the membrane enriched fraction of cardiomyocytes. n=4-5. **B)** The cell-permeable Ca^2+^ chelator, BAPTA-AM, prevents miR-204 mimic-induced decrease in APJ expression in the membrane enriched fraction of cardiomyocytes. n = 3-5. **C)** The Z-stack of cardiomyocytes showing the effect of miR-204 mimic and BAPTA-AM on Rab5a (green) and APJ (red) colocalization in three-dimension. The x, y, and z-axis are indicated. In control (SC) cardiomyocytes, APJ positive signal is localized near the nucleus. MiR-204 mimic-treated cardiomyocytes have the formation of Rab5a positive puncta, which colocalize with the APJ puncta. The addition of BAPTA-AM decreases the miR-204 mimic-induced Rab5a puncta formation and its colocalization with APJ in the cardiomyocytes. **D)** The quantification and representative image for Ca^2+^ (Fluo-3 AM) in H9C2 cells 24 hours after transfection with either SC or miR-204 mimic. Magnification ×20. Data are shown as mean, and error bars represent s.e.m. *p<0.05, **p<0.01 vs. indicated group. SC; scrambled control, miR-204 M; miR-204 mimic, B; BAPTA-AM.

**
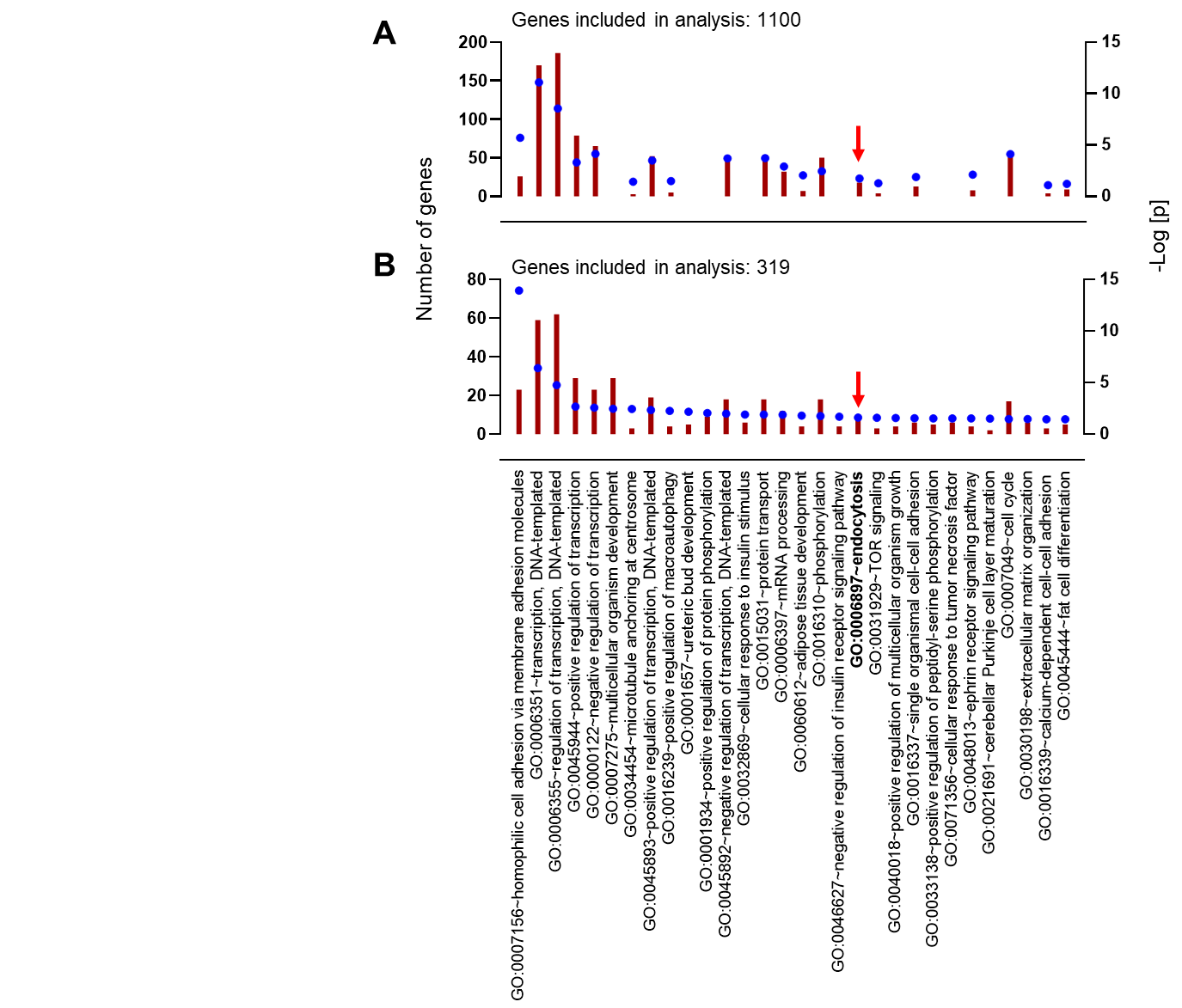
**

**Figure S8. The gene-ontology analysis of miR-204 target genes expressed in the heart.** The gene-ontology enrichment analysis using the Database for Annotation, Visualization, and Integrated Discovery (DAVID ) v6.8 shows the top 30 biological processes changed by the genes expressed in the heart and targeted by the miR-204. **(A)** Includes miR-204-target genes with SVR score ≤ -0.3 (1100 genes). **(B)** Includes miR-204-target genes with SVR score ≤ -0.9 (319 genes).

**
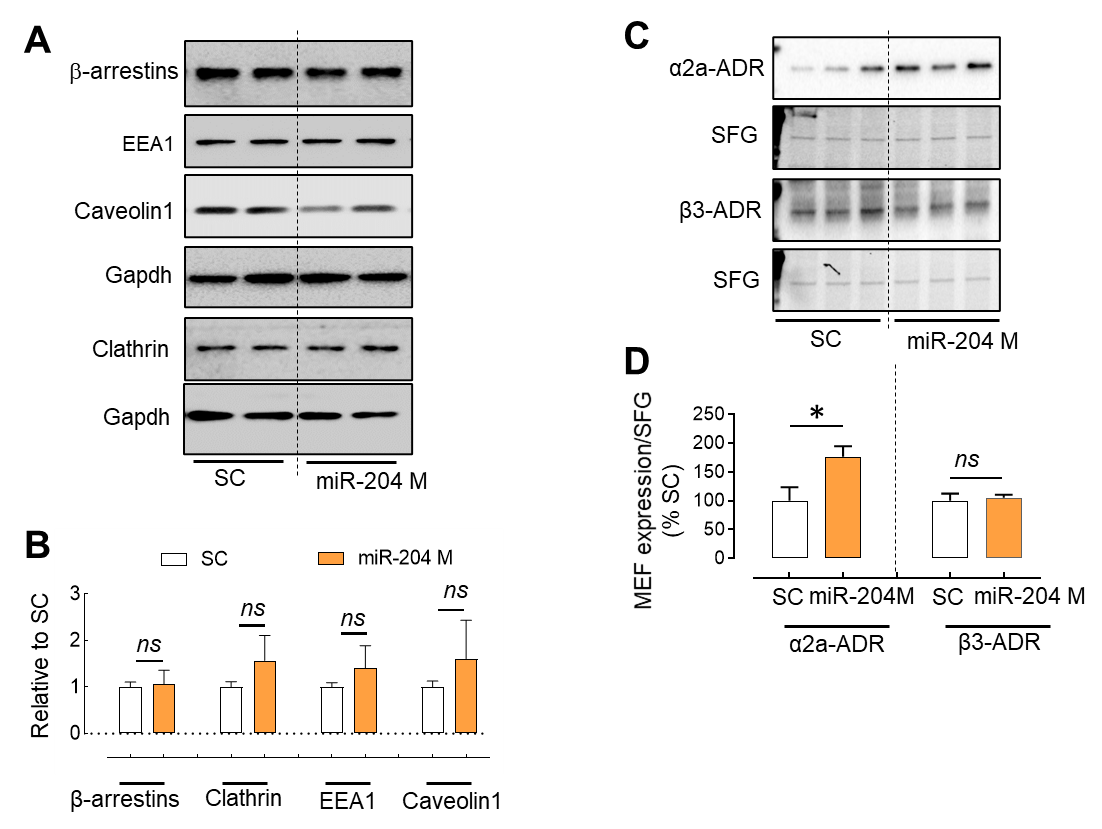
**

**Figure S9. Effect of miR-204 on the expression of β-arrestins, EEA1, caveolin-1, and Clathrin. A)** The effect of miR-204 mimic on the expression of endocytosis mediators (β-arrestins, EEA1, Cav1, and Clathrin) in H9C2 cells. **B)** Quantification of proteins in "A." n = 4-5. **C)** Immunoblots showing the effect of miR-204 mimic on the membrane expression of the α2a-adrenergic receptor (α2a-ADR) and β3-adrenergic receptor (β3-ADR) in HEK293 cells. **D)** Quantification of membrane α2-ADR and β3-ADR expression in "C". n=6. ns; not significant, *p<0.05, vs. indicated group. SC; scrambled control, miR-204 M; miR-204 mimic, EEA1; early endosome-associated protein 1, Cav1; caveolin 1, α2a-ADR; alpha2a adrenergic receptor, β3-ADR; beta3-adrenergic receptor.

**
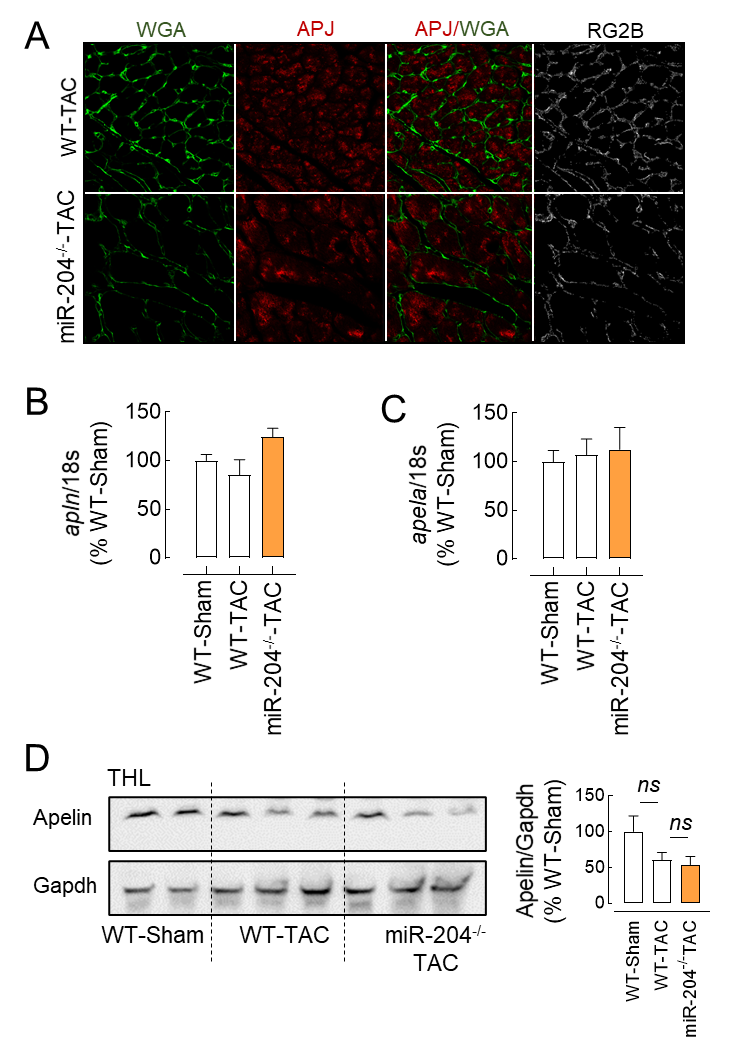
**

**Figure S10. A)** Representative image showing the WGA and APJ expression in the cardiac sections of wildtype-TAC and miR-204^-/-^TAC mice. Colocalization of APJ with WGA was determined using the RG2B ImageJ plugin (magnification ×63). **B & C)** The expression of *Apln* (gene encoding apelin) and *Apela* (gene encoding elabela) in the heart of WT-Sham, WT-TAC, and miR-204^-/-^ TAC mice. **D)** The immunoblot shows the expression of apelin in the heart of WT-Sham, WT-TAC, and miR-204^-/-^ TAC mice and its quantification. n = 4. Data are shown as mean, and error bars represent s.e.m. ns; not significant. THL; Total heart-tissue lysate.

**
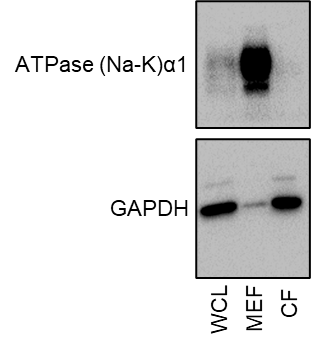
**

**Figure S11. Purity of membrane-enriched fraction.** The measurement of ATPase (Na-K) α1 expression in the whole cell lysate (WCL), the membrane-enriched fraction (MEF), and a cytoplasmic fraction (CF) of H9C2 cells.

| **Table-S1. Echocardiographic parameters of WT and miR-204^-/-^ mice.** | | | | |
| --- | --- | --- | --- | --- |
| **Parameter** | **Sham** | | **TAC** | |
|  | **WT** | **miR-204^-/-^** | **WT** | **miR-204^-/-^** |
| LV wall thickness (mm) | 0.82 ± 0.03 | 0.82 ± 0.03 | 1.09 ± 0.03^***^ | 1.20 ± 0.03^#^ |
| EF (%) | 86.98 ± 1.10 | 82.88 ± 1.38 | 63.12 ± 7.31^*^ | 55.41 ± 9.39 |
| FS (%) | 65.50 ± 3.50 | 58.46 ± 1.64 | 44.60 ± 8.47 | 35.67 ± 6.99 |
| HR (BPM) | 629.0 ± 11.55 | 667.56 ± 20.47 | 587.67 ± 22.60 | 490.38 ± 41.37^#^ |
| EDV (µL) | 38.10 ± 3.05 | 30.14 ± 3.14 | 48.49.14 ± 7.09 | 76.58 ± 10.04^#^ |
| ESV (µL) | 5.03 ± 0.70 | 4.88 ± 0.57 | 15.99 ± 5.42 | 43.12 ± 12.90^#^ |
| GLS (%) | -14.42 ± 2.06 | -12.20 ± 1.12 | -9.04 ± .98^*^ | -7.16 ± 1.15^#^ |
| PRS (%) | 58.27 ± 1.30 | 45.56 ± 0.32 | 37.24 ± 1.40 | 35.22 ± 2.73 |
| LV; Left Ventricular, EF; Ejection Fraction, FS; Fractional Shortening, HR; Heart Rate, EDV; End Diastolic Volume, ESV; End Systolic Volume, GLS; Global Longitudinal Strain, PRS; Peak Radial Strain. Data are shown as mean, and the error bar represents s.e.m. *p < 0.05 and ***p < 0.001 vs. WT Sham. ^#^p < 0.05 vs WT TAC. | | | | |

| **Table-S2. Echocardiographic parameters of miR-204^-/-^ TAC mice receiving AAV9-Control or AAV9-miR-204 virus.** | | |
| --- | --- | --- |
| **Parameter** | **miR-204^-/-^ TAC** | |
|  | **AAV9-Control** | **AAV9-miR-204** |
| LV wall thickness (mm) | 1.15 ± 0.03 | 0.97 ± 0.03^**^ |
| EF (%) | 83.70 ± 1.11 | 88.79 ± 0.53^**^ |
| FS (%) | 63.22 ± 1.93 | 70.52 ± 2.29^*^ |
| HR (BPM) | 559.44 ± 16.05 | 639.60 ± 10.05^**^ |
| EDV (µL) | 28.83 ± 1.80 | 21.77 ± 1.11^*^ |
| ESV (µL) | 4.48 ± 0.42 | 2.44 ± 0.26^*^ |
| GLS (%) | -4.25 ± 0.20 | -10.66 ± 3.43 |
| PRS (%) | 21.77 ± 2.09 | 37.58 ± 2.62^*^ |
| LV; Left Ventricular, EF; Ejection Fraction, FS; Fractional Shortening, HR; Heart Rate, EDV; End Diastolic Volume, ESV; End Systolic Volume, GLS; Global Longitudinal Strain, PRS; Peak Radial Strain. Data are shown as mean, and the error bar represents s.e.m. *p < 0.05, **p < 0.01 vs. miR-204^-/-^ TAC AAV9 Control. | | |

| **Table-S3. List of primers and their sequences** | | |  |
| --- | --- | --- | --- |
| **mRNA** | **Primer Sequence** |  |  |
|  | **Forward** | **Reverse** |  |
| *Nppb (mouse)* | 5’-CTC AAG CTG CTT TGG GCA CAA GAT-3’ | 5’-AGC CAG GAG GTC TTC CTA CAA CAA-3’ | |
| *Nppb (rat)* | 5’-TTT GGG CAG AAG ATA GAC CG-3’ | 5’-AGA AGA GCC GCA GGC AGA G-3’ |  |
| *Nppa (mouse)* | 5'-ATT GAC AGG ATT GGA GCC CAG AGT-3' | 5'-TGA CAC ACC ACA AGG GCT TAG GAT-3' |  |
| *Nppa (rat)* | 5'-GCC GGT AGA AGA TGA GGT CAT G-3' | 5'-GCT TCC TCA GTC TGC TCA CTC-3' |  |
| *β-mhc (rat & mouse)* | 5’-GAC AAC GCC TAT CAG TAC ATG-3’ | 5’-TGG CAG CAA TAA CAG CAA AA-3’ |  |
| *Aplnr (mouse & rat)* | 5'-CCA CCT GGT GAA GAC TCT CTA CA-3' | 5'-CTG ACG TAA CTG ATG CAG GTG-3' |  |
| *Sirt1 (mouse)* | 5'-AAT GCT GGC CTA ATA GAC TTG CA -3' | 5'-CCG TGG AAT ATG TAA CGA TTT GG-3' |  |
| *Col1a1 (mouse)* | 5’-GCC CAC AGC CTT CTA CAC-3' | 5’-CCA GGG TCA CCA TTT CTC-3' |  |
| *Col1a2 (mouse)* | 5’-GAC GCC ATC AAG GTC TAC TG-3' | 5’-ACG GGA ATC CAT CGG TCA-3' |  |
| *Col3a1 (mouse)* | 5’-GGA GGG AAC GGT CCA CGA T-3’ | 5’-GAG TCC GCG TAT CCA CAA-3’ |  |
| *Apln (mouse)* | 5'-CCTTGACTGCAGTTTGTGGA-3' | 5'-CTCGAAGTTCTGGGCTTCAC-3' |  |
| *Apela (mouse)* | 5'-CAC ACA CAT ACC TTC CCC TTT CT-3' | 5'-CAA AAC TGC CCA GGT GAA TAA AA-3' |  |
| *18S (rat, mouse & human)* | 5’-GCC GCT AGA GGT GAA ATT CTT A-3' | 5’-CTT TCG CTC TGG TCC GTC TT-3' |  |
| **microRNA** | **Sequence** |  |  |
| miR-204-5p | 5’-UUC CCU UUG UCA UCC UAU GCC U- 3’ |  |  |
| **microRNA** | **Primer Sequence** |  |  |
| miR-204-5p | 5’-CGC TTC CCT TTG TCA TCC TA-3' |  |  |
| RNU6 | 5’-GCA AAT TCG TGA AGC GTT CC-3' |  |  |
| **Modulators** | **Sequence** |  |  |
| Scrambled control | 5’-ACG TCT ATA CGC CCA- 3' |  |  |
| microRNA-204-5p inhibitor | 5’-AGG ATG ACA AAG GGA-3’ |  |  |
| **Genotyping** | **Primers Sequence** |  |  |
|  | **Forward** | **Reverse** |  |
| miR-204 | 5’-AAT GCTGGT CAG TGG CTA AGA-TGC-3’ | 5’-AGG AAA GTT ATG GGC TCA ATG ATG G-3’ |  |


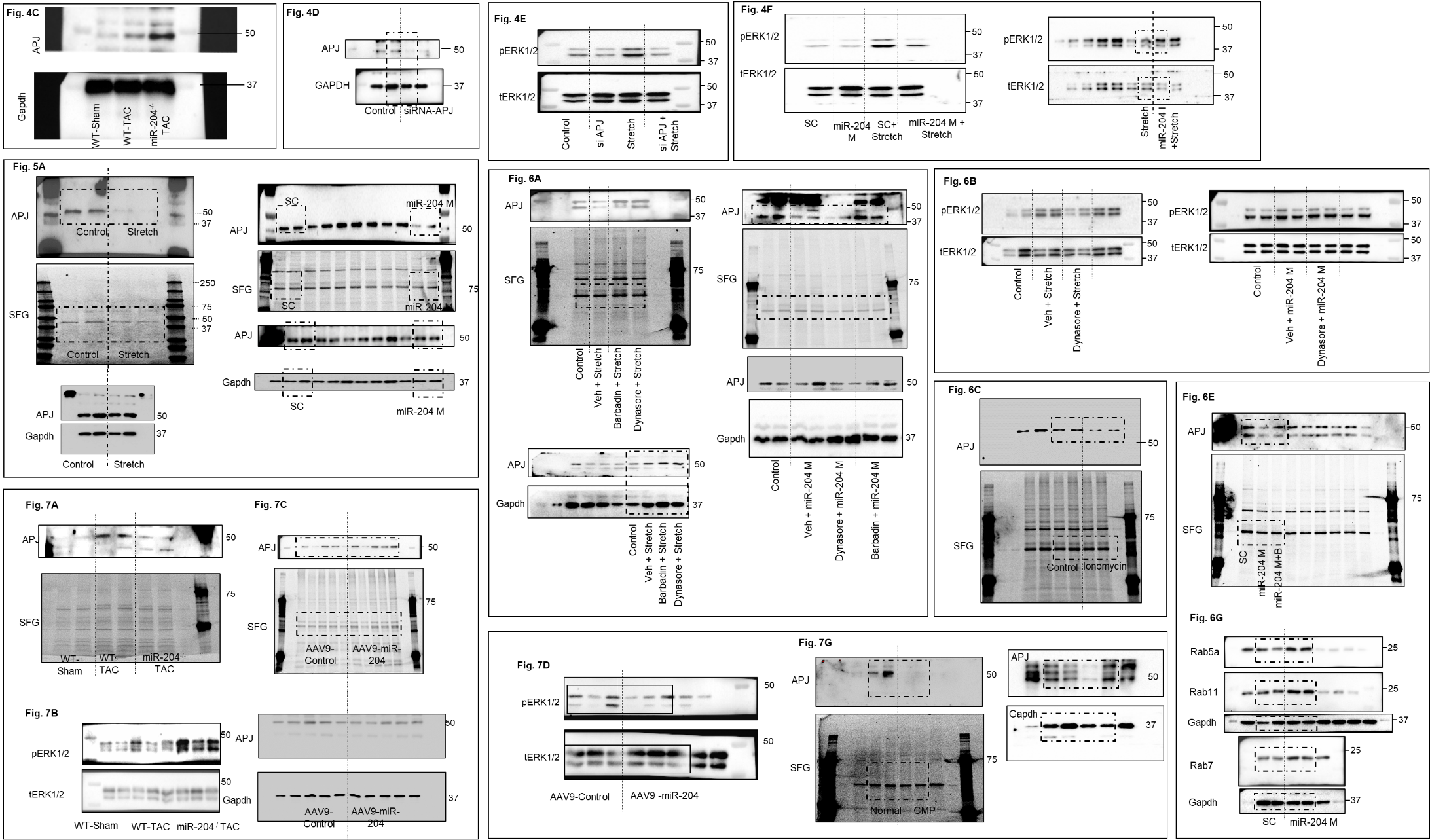


**Figure S12. Uncropped blot for the main figures.**


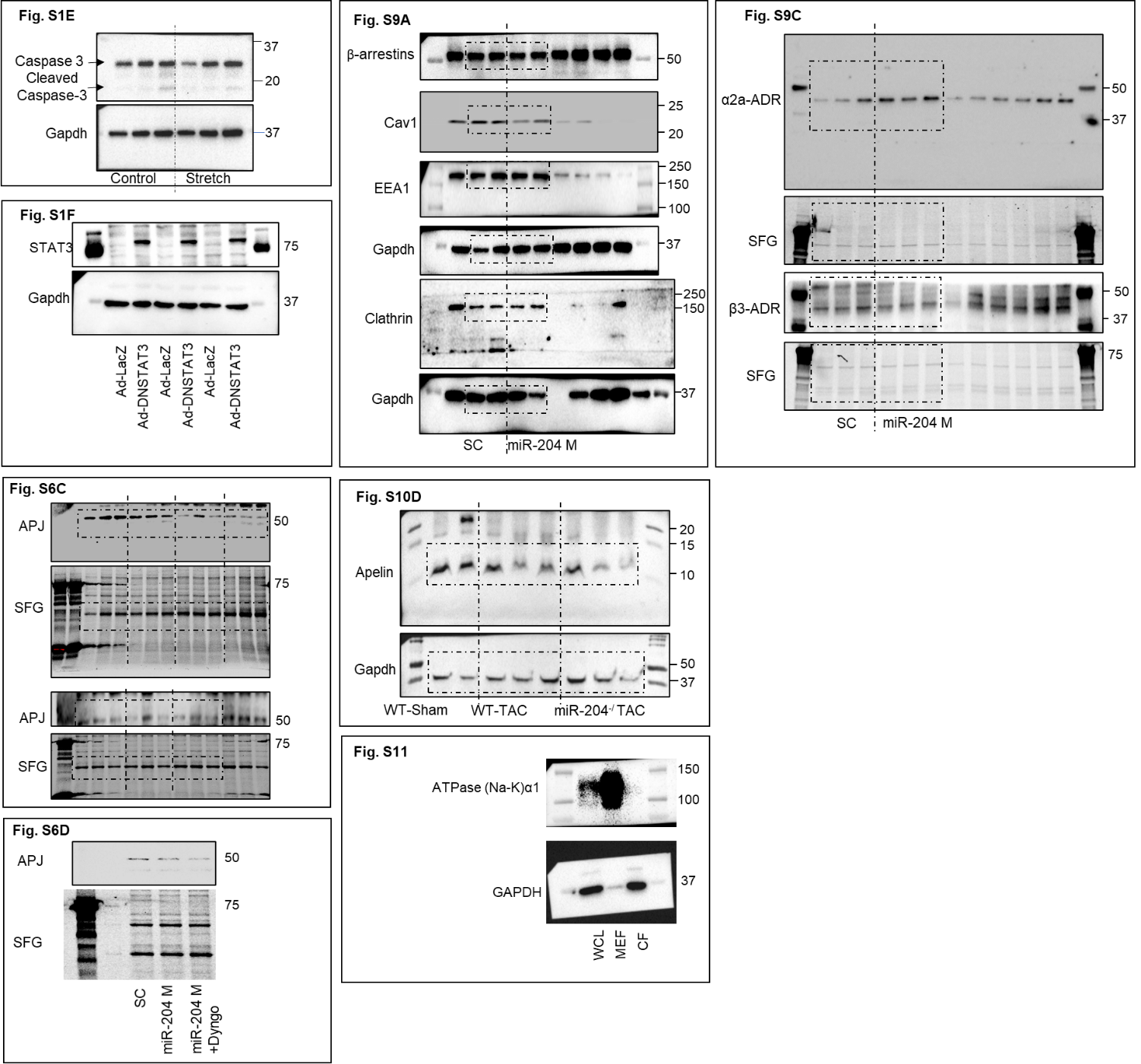


**Figure S13. Uncropped blot for the supplemental figures.**
